# Deep learning reveals genomic regions introgressed between two recurrently hybridizing lynx species

**DOI:** 10.1093/molbev/msag086

**Published:** 2026-04-08

**Authors:** Enrico Bazzicalupo, Lorena Lorenzo-Fernández, Lucía Mayor-Fidalgo, Laura Soriano, Daniel R Schrider, José A Godoy

**Affiliations:** Estación Biológica de Doñana, CSIC, Seville, Spain; Estación Biológica de Doñana, CSIC, Seville, Spain; Estación Biológica de Doñana, CSIC, Seville, Spain; Estación Biológica de Doñana, CSIC, Seville, Spain; Department of Genetics, School of Medicine, University of North Carolina, Chapel Hill, NC, USA; Estación Biológica de Doñana, CSIC, Seville, Spain

**Keywords:** population genomics, introgression, deep learning, Eurasian lynx, Iberian lynx

## Abstract

Recently, diverged species with overlapping distributional ranges have high chances of hybridizing and if hybrids are viable, genomic material can be transferred between species in a process called introgression. To characterize the patterns and consequences of introgression in species with historically low population sizes and recent steep declines resulting in genetic erosion, we analyze the Iberian and Eurasian lynx (EL) as an illustrative and relevant case study. While genome-wide introgression was already detected, here we apply a method using a deep convolutional neural network to detect specific regions of the genome with signals of introgression in three populations of these two species. Over 6% of the genome of both Iberian lynx and ELw shows introgression from the other species, compared with only 2% in the ELs. This observation, along with the results from demographic modeling, suggests that the ELw population is genetically closest to the source of EL introgression, a probably now extinct group that coexisted with the Iberian lynx in Southern Europe and Northern Iberia until recently. As predicted by theory, introgression was generally higher in populations with smaller effective sizes and in genomic regions of high recombination. However, the Iberian lynx did not show higher overall introgression than the more abundant ELw, and coding regions introgressed as frequently as intergenic regions. Local genetic diversity is boosted approximately 3-fold in genomic windows where introgression occurs, potentially including the adaptively relevant and highly diverse MHC region of the Iberian lynx.

## Introduction

Introgression is the incorporation of genetic material from one species into another, achieved via recurrent backcrossing of hybrids with one of the parent species. Introgression between divergent lineages is a relatively common process across the tree of life, and examples of interspecific introgression have been discovered in a wide variety of taxonomic groups (Schardl and Craven 2003; Gladieux et al. 2014; Nadeau et al. 2014; Goulet et al. 2017; Li et al. 2019; Vanderpool et al. 2020; Suvorov et al. 2022). Introgression moves alleles across species boundaries, counterbalancing the divergence resulting from both neutral and adaptive forces (Lenormand 2002; Yeaman and Otto 2011). Although introgression between distantly related species is not impossible, introgression is more likely to occur between recently diverged lineages that have accumulated fewer hybrid incompatibilities (Matute et al. 2010). For introgression to occur, the two species must also present overlapping distributional ranges, where the hybridization events can happen (Barton and Hewitt 1989). Although limited by geographic and genetic distances among species, a large fraction of closely related species hybridizes in nature (Mallet 2005).

Introgression can have both beneficial and harmful effects on the recipient population. On average, introgressed alleles located in genes and other functional genomic regions should negatively impact the fitness of the receiving population, either because of the presence of accumulated genomic incompatibilities, or because they are maladapted in their new environment (Matute et al. 2010; Arnegard et al. 2014; Lindtke and Buerkle 2015; Harris and Nielsen 2016). Nevertheless, introgressed alleles might reduce the expression of fully or partially recessive deleterious mutations in heterozygotes resulting in heterosis (Ingvarsson and Whitlock 2000). Threatened populations that have faced dramatic population declines are likely to benefit the most out of the potential fitness increases associated with introgression, resulting in genetic rescue (Whiteley et al. 2015) and high effective migration rates (Ingvarsson and Whitlock 2000; Bierne et al. 2002; Moran et al. 2021). On the other hand, introgression can also represent a significant risk for the long-term survival of endangered species, by threatening extinction by population replacement and genetic swamping (Todesco et al. 2016; Harris et al. 2019), or by introducing maladaptive or novel deleterious alleles (ie genetic load) which, when recessive, are subsequently exposed by inbreeding (Kyriazis et al. 2021). To effectively conserve and manage threatened populations experiencing introgression, it is therefore essential to determine whether the benefits of gene flow outweigh its potential negative consequences. This requires assessing the extent to which a threatened population has experienced, or is currently experiencing, introgression, identifying the source populations contributing to gene flow, and evaluating the genomic consequences of introgression. Such evaluation includes determining which genomic regions are affected and how introgression influences key genetic indicators, including genetic diversity.

**Figure 1:**
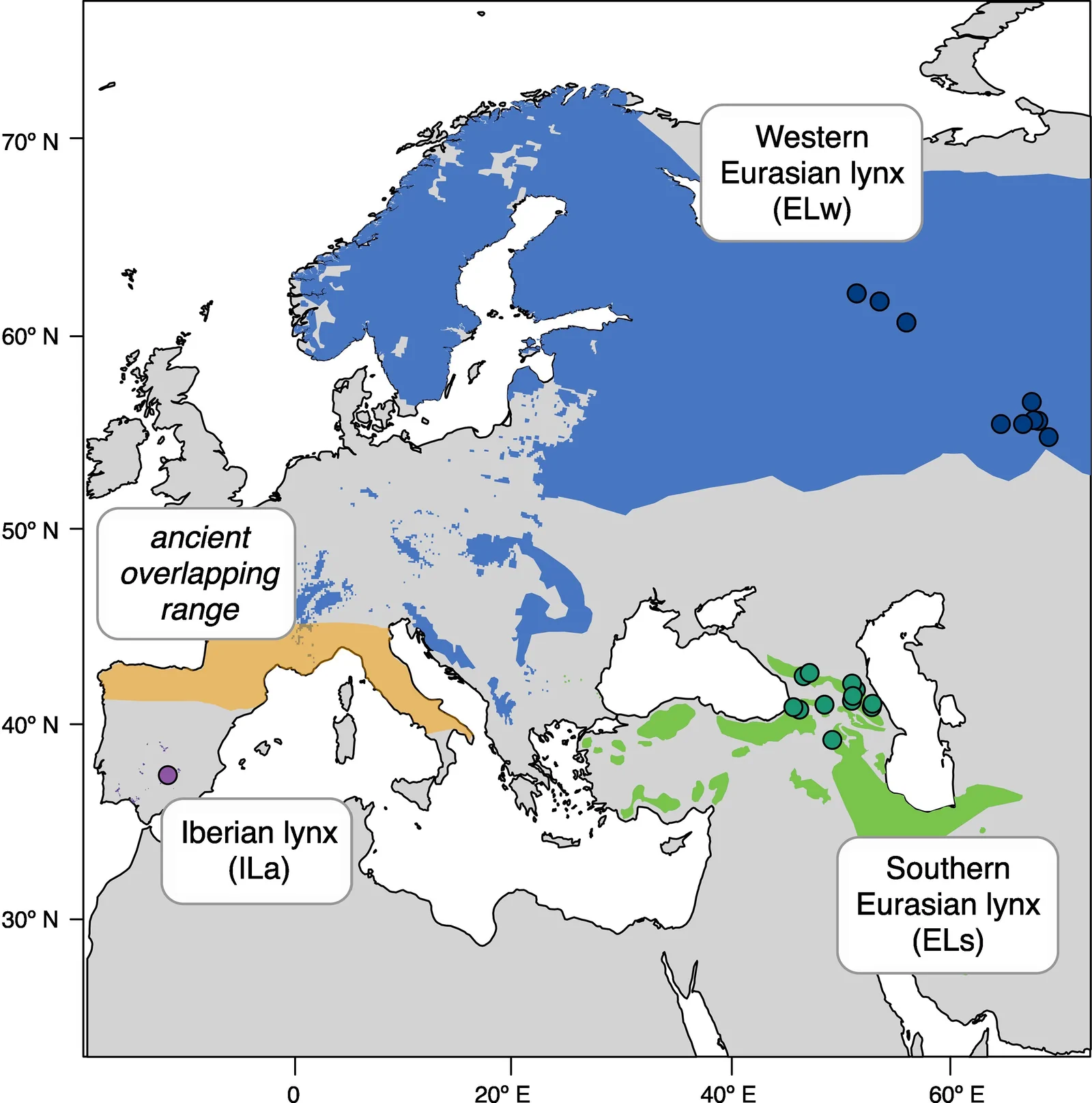
Distributional ranges of the three lynx populations: Iberian lynx (ILa), Western Eurasian lynx (ELw), and Southern Eurasian lynx (ELs). Supposed ancient range overlapping region where the two species coexisted is also highlighted. Sampling locations for the sequenced individuals are marked by circles and color coded by population. It must be noted that European populations west of the Carpathians, including the Alpine, Central Europe, and Dinaric regions, are reintroduced. Spatial geographic ranges were downloaded from IUCN (Breitenmoser et al. 2015; Rodriguez 2024).

The Eurasian lynx (EL), *Lynx lynx* (Linnaeus, 1758), and the Iberian lynx, *Lynx pardinus* (Temminck, 1827) are two closely related and geographically proximate species that exhibit unique ecological adaptations and contrasting demographic histories. The Iberian lynx is a medium-sized carnivore endemic to the Iberian Peninsula (Rodriguez 2024). During the last century, severe population declines caused by habitat loss, hunting, and prey-depletion left the species with less than a hundred total individuals divided into two small remnant populations in the southern part of the peninsula (Rodríguez and Delibes 2002; Casas-Marce et al. 2017). Historical and ancient distributions of the species were much wider, with both fossil and molecular evidence showing that the Iberian lynx's range included most of the Iberian Peninsula and extended to the southern part of France and Italy in the early Pleistocene (Kurtén and Granqvist 1987; Rodríguez-Varela et al. 2015; Mecozzi et al. 2021). The Iberian lynx population later began to contract, and its distribution was already largely restricted to the southwestern quadrant by the 16th century until its dramatic decline during the 20th century (Rodríguez and Delibes 2002; Clavero and Delibes 2013; Abascal et al. 2016; Casas-Marce et al. 2017). In contrast, the EL is a widespread carnivore whose distributional range extended across most of Eurasia during the Holocene, although its range has also been dramatically reduced in Western Europe since the 16th century, leaving only populations in the Carpathian Mountains, the Baltic states, and the Balkans (Breitenmoser et al. 2015). Recent whole genome sequencing studies have found that at least three distinct nuclear clades have formed in this species’ range (Lucena-Perez et al. 2020; Mengüllüoğlu et al. 2021; Bazzicalupo et al. 2022). One clade inhabits the eastern part of the distribution, separated by the Urals Mountain range from a distinct clade in the west, while a southern clade is separated from both by a discontinuous geographic range and the Himalayan Mountains. The western clade appears to have suffered the most dramatic declines in population sizes during the last century, with habitat-loss driving local extinction and fragmentation of most of the European populations (Lucena-Perez et al. 2020). Although currently the westernmost remnant population of EL inhabits the Carpathian Mountains (Breitenmoser et al. 2015), in ancient and historical times the species coexisted with the Iberian lynx in Southern France, Italy, and even in the northern part of the Iberian Peninsula until as recently as 200 years ago (Clavero and Delibes 2013; Rodríguez-Varela et al. 2016; Lucena-Perez et al. 2022) (Fig. 1).

Divergence between the Eurasian and Iberian lynx recovered from molecular data is relatively low. Early phylogenomic analyses estimated a divergence time of around 1 million years, placing them as sister species within the *Lynx* clade (Johnson 2004; Johnson et al. 2006; Li et al. 2016a). Although this topology is the most commonly accepted phylogeny and the one most frequently reconstructed along the genome, an alternative topology including a sister relationship between the Canadian and EL and an older divergence of Iberian lynx is reconstructed from low-recombination regions of the autosome and X chromosome (Li et al. 2019)—regions, which are expected to yield more accurate species tree inferences in the presence of incomplete lineage sorting (ILS) or introgression (Pease and Hahn 2013). This suggests the tree with Canadian and EL as sister species is more likely to be the true topology, and support for the alternative Iberian–Eurasian topology may in part be the result of extensive introgression happening between Iberian and EL after their divergence (Li et al. 2019). This gene flow may have been recent, as higher levels of introgression from EL were estimated from contemporary Iberian lynx genomes than ancient ones dated between two and four thousand years ago (Lucena-Perez et al. 2024). This recent introgression could explain the increased genetic diversity observed in the contemporary Iberian lynx population relative to ancient genomes. Demographic reconstructions based on sequential Markov coalescence models also found a model with postdivergence migration more likely than a simpler isolation model, with complete isolation estimated to have occurred only very recently (Abascal et al. 2016).

Although these recent studies have provided compelling evidence of postdivergence gene flow between Iberian and EL, some questions are left unanswered. Overall, the analyses performed to date strongly support recent introgression between the two species, but do not have the power of estimating the amount of introgression in each species genome. The D-statistic results of Li et al. (2019) cannot be used to estimate the amount or directionality of gene flow (Hibbins and Hahn 2022). The introgressed fraction and directionality estimated in Lucena-Perez et al. (2024) were limited by the comparison between ancient and contemporary Iberian lynxes, and did not tackle the entire history of gene flow between the two species. Even when migration rates through time were estimated by Abascal et al. (2016), they were assumed to be symmetrical and constant. Additionally, all of these analyses have provided genome-wide tests or estimates, without delimiting introgressed regions along the genome. Most methodological approaches aimed at finding introgression in genomic windows have either relied on panels of reference “pure” individuals free of admixture (Brisbin et al. 2012; Lawson et al. 2012), or by using one or more summary statistics designed to detect the signature of introgression (Geneva et al. 2015; Rosenzweig et al. 2016; Schrider et al. 2018; Durvasula and Sankararaman 2019). A more recent and promising approach to detect local patterns of introgression is based on using genomic variants as a direct input to deep convolutional neural networks (CNN) (Ray et al. 2024), an approach that has proved successful for an array of different tasks in population genomics (Chan et al. 2018; Flagel et al. 2019; Smith et al. 2023). For example, Ray et al. (2024) were able to train a CNN to detect regions of introgression between two *Drosophila* species with high accuracy and use a separate fully CNN to accurately identify introgressed haplotypes and the genomes containing them. This supervised learning framework overcomes several limitations of alternative approaches in the task of detecting introgression between our study species. Primarily, its simulation-based training does not require reference individuals free of admixture, which are difficult to identify in this species pair due to recurrent gene flow among the potential ancestors of all extant populations. Additionally, many methods rely on in-group–out-group comparisons to detect introgression, an approach that is problematic given the unresolved species phylogeny and the possible sister relationship between the two species.

In this study, we aim to gain a more comprehensive understanding of the long-term genomic consequences of interspecific gene flow in threatened species and to address the knowledge gaps left by previous analyses by: (i) inferring the extent and reconstructing the genomic landscape of introgression in Iberian and EL genomes, and (ii) identifying the most likely source population of the Iberian lynx introgression. To do so, we applied the approach developed by Ray et al. (2024) to find genomic windows with signals of introgression among three Iberian and EL populations. Once we identified these regions in each species, we estimated the amount of introgression observable in each species’ genome, across different populations and genomic regions. We addressed the question of which of the remnant EL lineages is more closely related to the extinct population that coexisted with the Iberian lynx in Northern Iberia and Southern Europe. Finally, by looking at the genomic regions where introgression is happening, we evaluated what areas of the genome are more permeable to introgression, the biological functions of introgressed genes and how introgression has shaped local patterns of genetic diversity in the different populations.

## Results

We analyzed whole genome sequencing data from one Iberian lynx population and two distinct EL populations. Of the two remnant Iberian lynx populations, we chose to analyze individuals from the Andujar population (ILa), as it maintained higher genetic diversity and represents the highest proportion of the species’ prebottleneck genetic diversity, in contrast with the Doñana population whose genetic diversity has been lost at high rates due to a long period of low effective population size in isolation (Abascal et al. 2016; Casas-Marce et al. 2017; Lucena-Perez et al. 2021; Kleinman-Ruiz et al. 2022). Individuals belonging to the Western (ELw) and Southern (ELs) clades of EL were selected, as these clades were identified as distinct intraspecific lineages by genomic data (Lucena-Perez et al. 2020; Bazzicalupo et al. 2022) and they represent the two clades geographically closest to the possible contact point between the two lynx species (Sommer and Benecke 2006; Fig. 1). By joining sequencing data from previously published studies with 10 newly sequenced Iberian lynx genomes (Methods, Table S1), we generated a dataset that comprised whole genome sequences of a total of 54 lynx individuals (ILa = 22, ELw = 20, ELs = 12; Fig. 1).

Our sequencing, read alignment, and variant calling efforts resulted in an initial set of 33,660,575 raw autosomal variants. We then removed indels, nonbiallelic SNP, SNP not meeting the quality thresholds and any SNP falling within low-complexity or repetitive regions of the reference genome. This left a set of 6,495,299 high-quality biallelic SNPs in our VCF file. The 15% missing data filter applied to each population resulted in a loss of around 3.9% of SNPs in Iberian lynx, 4.2% in Western and 1.8% in the ELs. The lower missing rate in ELs is probably given by the relatively high sequencing depth and quality obtained for all of the samples we have from this population, while other populations contain a mix of individuals with both relatively high and relatively low sequencing depth. After removing SNPs from possible collapsed paralogs in each population, we generated datasets for each pair of Eurasian and Iberian lynx populations, obtaining 4,777,474 SNPs that were segregating in the Iberian–Western Eurasian (ILa-ELw) pair and 4,753,415 SNPs in the Iberian–Southern Eurasian (ILa-ELs) pair.

### Demographic inference

**Figure 2:**
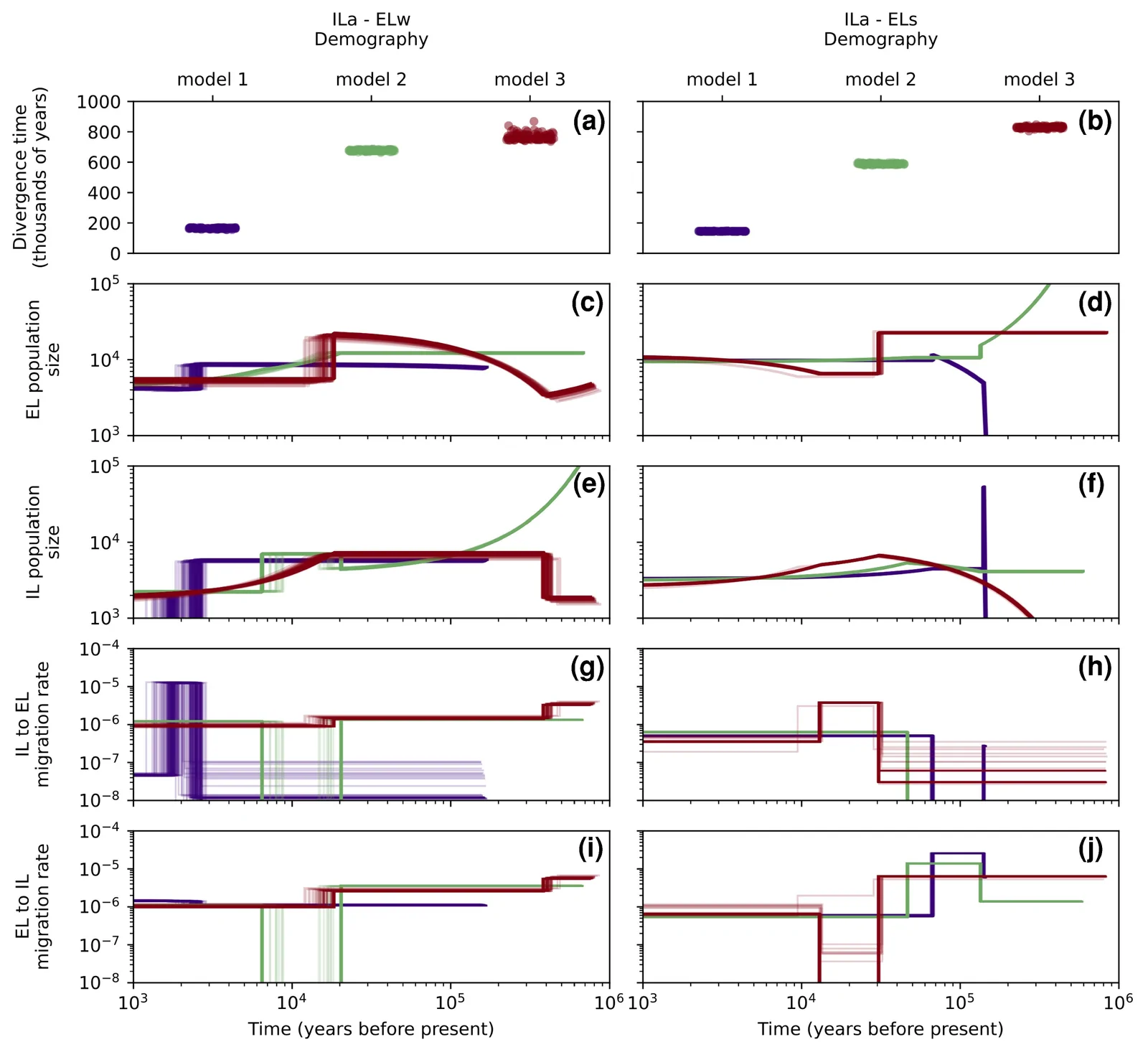
Summary of demographic modeling performed by running GADMA2's structure model with three postdivergence epochs. Out of 500 runs, the best three scoring models are represented for each population pair, ILa-ELw on the left and ILa-ELs on the right. Each dot or line represents the optimized value of the parameter for one of one hundred bootstrap replicate datasets. a, b) Divergence times, represented by dots, color coded by model. c–j) Lines follow the same color code and represent changes in effective population size (c–f) and directional migration rates (g–j) in the last million years, across the three postdivergence epochs of all the models. Time is transformed to years assuming a generation time of 5 years (Lucena-Perez et al., 2018). Migration rates are defined in forward time as the probability of choosing a parent in the sink population from the source population. All values, except for the divergence times on top, are represented on a logarithmic scale.

As the methodological approach relies on training a model based on simulated data under different introgression scenarios, we first performed demographic modeling between the two-population pairs, with the aim of both unraveling the recent and past demographic histories of these populations with a greater detail allowed by newly sequenced individuals and high-quality reference genomes, and creating a training dataset that was most similar to real data as possible. We sought to minimize any bias in our demographic inferences caused by natural selection by removing SNPs potentially affected by direct or linked selection (Schrider et al. 2016; Johri et al. 2021). We therefore removed SNPs within genes and those with missing data, and then pruned SNPs within 50 kbp of each other, resulting in a set of 31,628 SNPs in the ILa-ELw dataset, and 31,882 SNPs in the ILa-ELs dataset. We then used the joint site frequency spectra from these SNPs to estimate demographic histories using the software GAMDA2 (Noskova et al. 2023). We modeled the demographic history of ILa-ELw and ILa-EEL 500 times for each population pair. From these, we selected each pair's three highest likelihood reconstructions (Figure S1) and ran local optimizations using 100 bootstrap datasets. The following parameters were optimized in each model: (i) the length in generations of each time interval (epoch) in the model, two before and three after divergence between populations; (ii) effective population sizes of each population at the start and at the end of each epoch (these were equal if size was constant in the model, but differed if size changed exponentially during that epoch); (iii) migration rates in each direction during each postdivergence epoch, calculated as the probability that a chromosome present in one population (destination) descended from a chromosome that was present in the other population (source) in the previous generation. Model generation times are converted into years given a generation time of 5 years (Lucena-Perez et al. 2018). Divergence times between Iberian and EL are consistent between the demographic histories estimated from both population pairs, with both having one model placing the divergence around 150 kya, one model around 600 kya and one model around 800 kya (Fig. 2a, b).

Population trajectories of ELw and ELs are different in all model reconstructions (Fig. 2c, d). The effective population size of ELw in the most recent epochs is consistently estimated to be around 4 to 6 k individuals and to have experienced a significant recent decline in all three ILa-ELw models (Fig. 2c), although the decline's timing and intensity varies among models, decreasing from a starting size of 10 to 20 k individuals at a time varying between 3 and 20 kya. The trajectory through time of the ELs population is much more variable across models (Fig. 2d), slightly increasing in the last 10,000 years in one model, and staying roughly constant during the last 50,000 years in the other two models. In all models, the most recent population size is approximately double that of ELw, around 10 k individuals.

The recent effective population size of the ILa population follows a similar trend in all models, both when reconstructed in conjunction with ELw and with ELs (Fig. 2e, f). In all cases, ILa experiences a decline, although this decline is inferred to be sharper (down to approximately one third of the size before the contraction) and more recent (between 3 and 20 kya) in models with ELw (Fig. 2e), and less intense (around a 50% reduction), and more prolonged (occurring over a period of between 30 and 50 kya), in models with ELs (Fig. 2f). Regardless of intensity and timing, all models recover a decline of the ILa effective population from around 5 to 7 k individuals in ancient times to < 3 k individuals in more recent times.

Migration rates in both directions are generally higher between ILa and ELw than between the former and ELs (Fig. 2g–j). In the ILa-ELw models 2 and 3, ancient migration rates were higher than during the most recent epoch, with model 2 having a period of no migration between the two populations from 20 to 6 kya (Fig. 2g, 2i). The ILa-ELw model 1, which presents the lowest recovered divergence time among the three, shows a different pattern. In this model, migration from ILa to ELw around 2 kya has a dramatic increase, rising to 10 times the levels recovered for the other models in recent times, followed by a decline to around 4% of the other model's migration rates (Fig. 2g). Migration from ELw to Ila, on the other hand, is increasing slightly in that same time period. Reconstructions of ancient migration rates between ILa and ELs are more consistent among models (Fig. 2h, j). Ancient rates from ILa to ELs are always lower than what is estimated for recent times. The opposite is inferred for migration rates from ELs to ILa, which are always declining from ancient to most recent times in all models.

### Introgression scans

#### Model performance

After obtaining the demographic models described above, we used them to generate training simulations and then train classifiers to detect introgressed windows for each population pair (Methods). The discriminator model was trained to analyze a tensor representing genotypes from a genomic window and assign probabilities to four possible scenarios explaining the resulting alignment: (i) “ELtoIL”—introgression from EL into Iberian lynx, (ii) “ILtoEL”—introgression from Iberian lynx into EL, (iii) “BiDir”—introgression in both directions between the populations, and (iv) “none”—no introgression observed.

We compared both models’ performances in terms of precision and recall using 1,000 additional simulations under each introgression scenario using different probability thresholds (*P*) to define introgressed windows (see Methods, Figures S2 and S3). As the threshold parameter *P* increases, both the ILa-ELw and ILa-ELs models become more precise in detecting introgression by reducing false positives, as the criteria for calling introgression becomes stricter (Figure S2). This comes at the cost of a decrease in recall due to an increase in false negatives, whose sharpest decline happens when increasing *P* from 0.9 to 0.95. The model's ability to detect the directionality of introgression events is also affected by the *P* threshold we select (Figure S3). As the criteria to call bidirectional introgression gets stricter with higher *P*, we observe increased model precision and decreased recall for bidirectional introgression in both ILa-ELw and ILa-ELs models. This is because although we are increasingly precise in calling bidirectional cases correctly, we require our model's summed probability of introgression (Prob(unidirectional) + Prob(bidirectional)) to exceed *P* in both directions for a region to be classified as experiencing bidirectional introgression (see Methods), and this becomes increasingly difficult as *P* increases. This can also be represented as decreased precision with increasing *P* for unidirectional cases (the first and third panels in Figure S3), whose increased false positives come from incorrectly assigned bidirectional cases. In the ILa-ELw model, both directions are affected equally by this, while in the ILa-ELs model most of the incorrectly assigned bidirectional cases are assigned the “ELtoIL” category, which is the only one experiencing a decline in precision with higher *P*. Again, the sharpest declines in precision for the unidirectional categories and recall for the bidirectional category happen when increasing *P* from 0.9 to 0.95.

Given these results, when applying the model to real data, we selected 0.9 as the initial *P* threshold for both models (Figure S4). This would guarantee high precision in detecting introgressed windows (∼95% according to our simulated test data), avoiding the sharpest declines in recall introduced at higher *P*. Precision in detecting introgression directionality with this threshold is also >90% for all directions in both models, except for “ELtoIL” in the ILa-ELs model, which is slightly lower (∼85%), given the higher proportion of “BiDir” windows inferred to be “ELtoIL.” When applied to real data, we would expect the ILa-ELw model to slightly underestimate the amount of introgression in both directions, as “BiDir” cases are equally likely to be lost to either direction. The ILa-ELs model, on the other hand, is expected to slightly underestimate the amount of introgression into ELs, as bidirectional introgressed is more likely to be estimated as introgression exclusively into ILa (“ELtoIL” cases). The initial value for *P* of 0.9 avoids sharper declines in both precision and recall in detecting introgression directionality as well. We additionally opted to extend this initial *P* to 0.7 for windows next to introgressed ones. The rationale being that because introgressed haplotypes may stretch across multiple windows, windows next to those affected by introgression are more likely to be affected by introgression themselves and thus should be subject to a more lenient threshold to further reduce false negatives.

With the optimal value for *P* selected, model performance was additionally evaluated with evaluation datasets aimed at emulating low average sequencing depths (between 2× and 6×) and considering more “realistic” data, including both the presence of some low-depth individuals and some variance in mutation and recombination rates. With a sequencing depth of at least 4× and with “realistic” dataset conditions, precision and recall values for all tasks are very similar and comparable to the model's performance on “ideal” data with no genotyping errors and no heterogeneity in mutation and recombination rates (Figures S5 and S6). Increasing sequencing depth has no effect on the precision of detecting the presence of introgression in a window, although it increases its recall, meaning a reduction in false negative introgression calls (Figure S5). Increased sequencing depth also has limited effect on the model's ability to detect the directionality of the introgression events within a window with introgression, mostly increasing the precision of “ELtoIL” calls and drastically improving the recall of bidirectional introgression detection (Figure S6). Overall, the sequencing conditions of our study appear to be sufficient to yield model performance similar to what we would obtain on perfect data, reflected by the close to identical scores of “realistic” and “ideal” evaluation sets for all classification tasks.

#### Introgressed windows and downstream analyses

**Figure 3:**
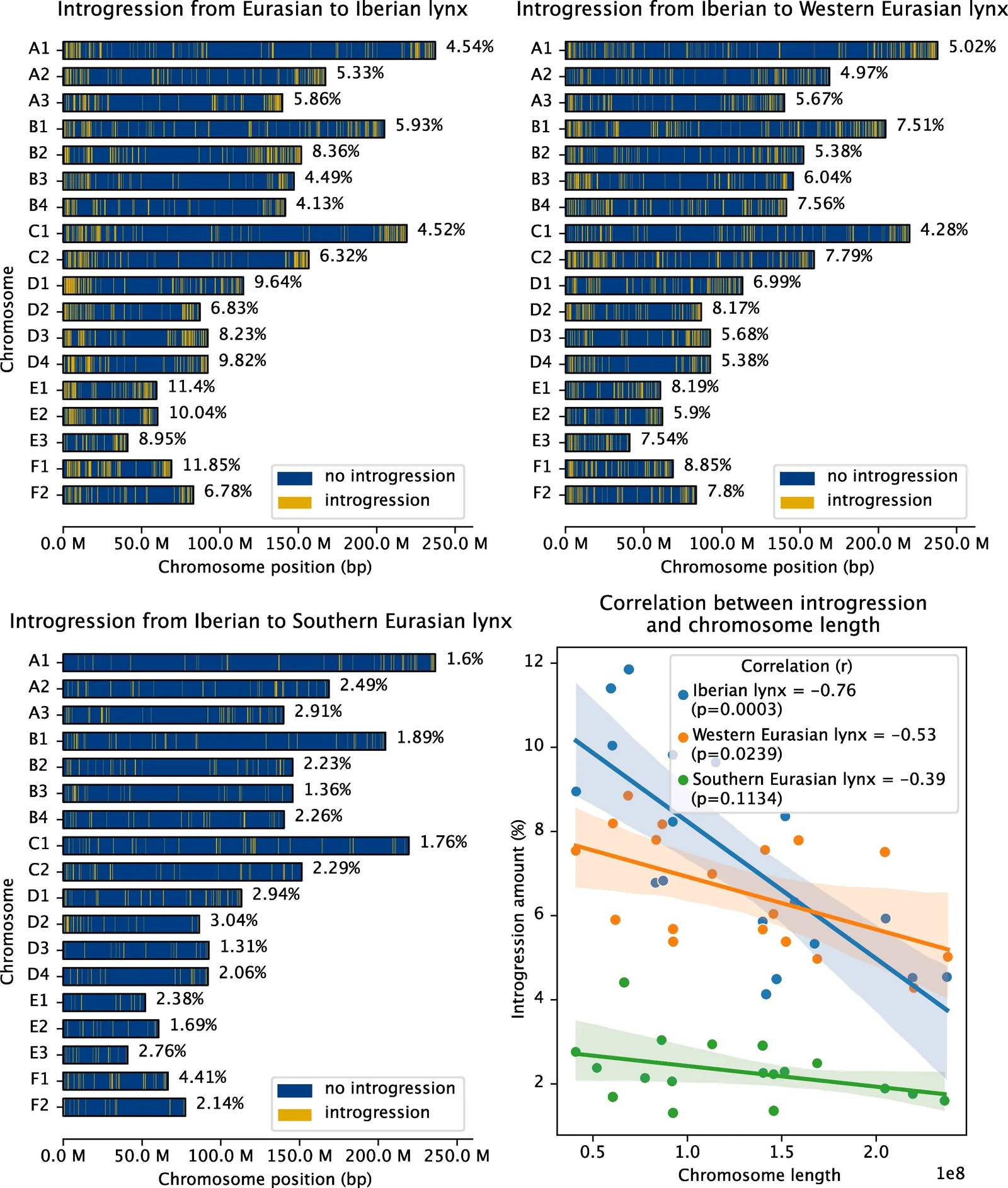
Introgression along the autosomes of the three lynx populations (top and bottom-left) and relationship between chromosome length and the amount of introgression (bottom-right). Each population's autosome is represented as a bar where introgressed windows are highlighted. Each chromosome's percentage of base-pairs occupied by windows with introgression is specified. Bottom-right panel shows the relationship between chromosome length (*x* axis) and introgression amount (*y* axis) in the three populations. A linear regression line with 95% confidence intervals shows the tendency of the relationship. Pearson's correlation coefficient (*r*) between the two values and its *P*-value are reported for each population.

We applied each model to its corresponding population-pair phased dataset, obtaining predictions of introgression presence and direction for each genomic window (Fig. 3). We classified a total of 1,517 distinct noncontiguous genomic windows, occupying a total of 6.24% of the genome, as experiencing introgression from the ILa population into ELw, and 519 genomic windows, occupying around 2.13% of the genome, as introgressed from ILa into ELs. Introgression into ILa was inferred to affect 5.55% of the genome (1,348 genomic windows) in the model with ELw and 4.60% (1,071 genomic windows) in the model with ELs. By taking the union of these two sets of introgressed windows, we obtain a total of 1,450 distinct genomic windows, for a total of 6.52% of the genome experiencing introgression from ELs to ILa, of which 1.92% was detected exclusively in the ELw-ILa comparison, 0.97% exclusively from the ELs-ILa comparison, and 3.63% detected from both. The average size of introgressed windows is similar in the genomes of the three populations, with median lengths of ∼70 kbp in all datasets and very low effect sizes calculated for differences among their distribution of values (Figure S7; Cohen's *d* (Cd) < 0.1). Introgression was observed in all the chromosomes of the three populations (Fig. 3). Chromosome F1 was consistently the one with the highest amount of introgression (11.85% in ILa, 8.85% in ELw, and 4.41% in ELs). Moreover, a negative correlation between the fraction of the chromosome affected by introgression and chromosome size was observed for the three lynx populations, although significantly so only for the genomes of ILa and ELw (Fig. 3).

Because mismatches between training and real data are always a concern when conducting simulation-based inference, we compared the joint site frequency spectrum (SFS) between observed genomic windows and those simulated for training purposes. This comparison revealed that, as expected under correct model predictions, observed windows inferred to result from a particular introgression scenario most closely resembled simulations incorporating that scenario into their demographic model (Figure S8). The amount of opposite-frequency shared variation (cells just below the top-left and just above the bottom-right corners of the SFS) is expected to be positively correlated with introgression amount. Accordingly, both simulated and observed windows without introgression show less of this type of variation than windows with introgression (Figures S9–S11). Although the observed windows with introgression show a lower average amount of opposite-frequency shared variation (and therefore introgression) than the simulations used for training (Figures S10–11), the broad range of introgression levels included in the training set should enable the discriminator model to correctly classify windows with a wide range of introgression amounts. Thus, while our training simulations are likely affected by some degree of misspecification, given that the SFS of regions classified as introgressed resemble simulated regions with introgression, our classifiers appear to behave as expected.

Genes found in the introgressed windows of the three populations were found to be enriched in a total of 64 distinct biological process (BP) GO terms encompassing a wide variety of different functions (Tables S2–S4). Genes introgressed into ILa are particularly enriched in GO terms related to the immune system, such as antigen interactions and humoral responses, and metabolic and catabolic pathways related to coenzyme A. Among enriched GO terms of genes introgressed into both ELw and ELs, we found abundance in functions related to tissue differentiation and development and neural structure and synaptic signaling.

**Figure 4:**
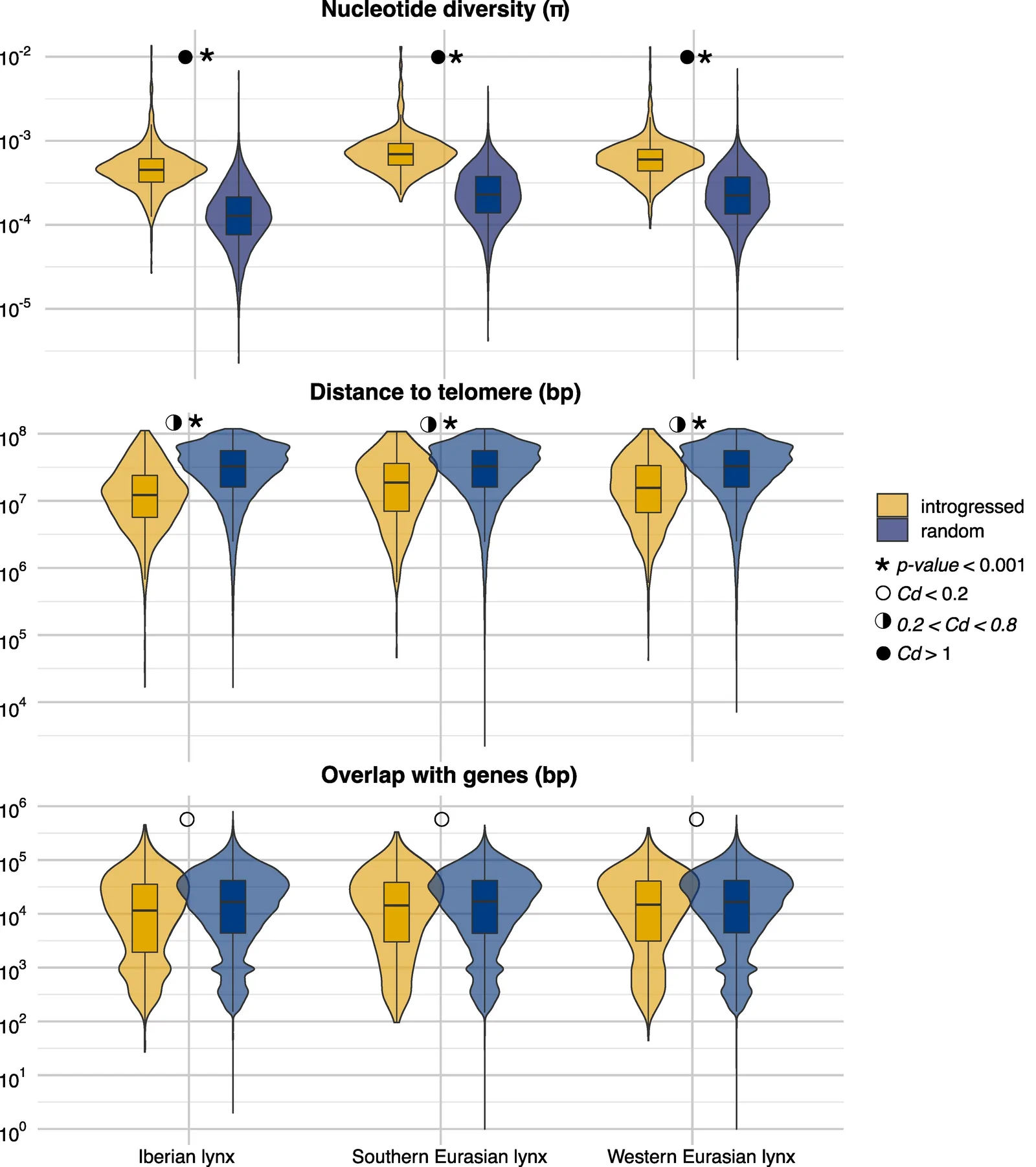
Violin and boxplots showing the distribution of nucleotide diversity (top), base-pair distance to closest telomere (middle), and number of base-pair overlap with genes (bottom) in windows with introgression and random subsamples of the genome for the three lynx populations. All values are represented on a logarithmic scale. Magnitude of effect size (Cd) and significance (*P*-value < 0.001) are represented above each comparison.

We explored the effects of introgression on the genetic diversity of the recipient population, and its interplay with recombination and selection by comparing nucleotide diversity (π), distance to telomeres (as a proxy of recombination) and gene content (as a proxy of selection) in introgressed windows and in random windows along the genome. This comparison revealed patterns which are consistent in all three lynx populations (Fig. 4). First, as expected, average π is significantly higher in windows with introgression relative to random windows without introgression (*P*-value ≪0.001 for all populations), with a relatively high calculated effect size (Cd) in all populations (ILa Cd = 1.81, ELw Cd = 1.57, and ELs Cd = 1.82). In terms of magnitude, ILa exhibits the greatest proportional increase, with introgression windows averaging roughly three and a half times the diversity of windows without introgression. Introgression windows in ELw and ELs have about two and a-half and three times as much diversity as windows without introgression, respectively. Introgressed windows also appear at higher densities near the ends of chromosomes (Fig. 4), with their average distance to telomere being significantly (*P*-value ≪0.001 for all populations) and moderately lower than that of random ones with no introgression (ILa Cd = 0.76, ELw Cd = 0.56, and ELs Cd = 0.49), with the lowest average distance observed in ILa. The amount of overlap with genes is very similar between random windows without introgression and introgressed windows in all populations (ILa: Cd = 0.09, *P*-value = 0.084; ELw: Cd = 0.12, *P*-value = 0.019; and ELs: Cd = 0.06, *P*-value = 0.44; Fig. 4).

## Discussion

### Phylogeography of introgression among lynx species

By applying a discriminator model trained to look at two-population genomic alignments and distinguish between introgression scenarios, we were able to detect windows with signals of introgression in the genomes of the three studied populations with high precision and recall. The Iberian lynx population of Andujar (ILa) and the Western Eurasian lynx population (ELw) appear to have accumulated the most introgression in their genomes: in both of these populations more than 6% of the genome shows signals of introgression from the other species. Much less introgression is observed in the Southern population of ELs, with signals of introgression found only in about 2% of its genome. Different rates of introgression from the two EL populations are also observed in the ILa genome. Although most of the introgression observed (3.6%) is found when looking at introgression with either population, more than two-thirds of the genomic regions detected from only one population pair were found in the ILa-ELw alignments (1.9% of the genome compared with 0.9% from ILa-ELs alignments).The difference in introgression levels between EL populations may reflect different levels of shared ancestry with the extinct population that inhabited the hybrid zone or different levels of recent gene flow with it. This is somewhat consistent with the close genetic relationship observed between westernmost remnant Carpathian and Baltic lynx populations with populations from the Western-Russia population used in our analysis, both in their nuclear and mitochondrial genomes (Mengüllüoğlu et al. 2021; Bazzicalupo et al. 2022). The lower levels of introgression in Southern lynx could also be compatible with no direct hybridization and indirect gene flow with the hybridizing population or its descendants. However, our results would also support the previous claim of a Holocene lineage shift in Southern Europe and Northern Iberia, where Southern-like mitochondrial clades from older specimens are replaced by Western-like mitochondrial clades in more recent specimens (Rodríguez-Varela et al. 2016; Lucena-Perez et al. 2022). In that context, it would make sense that both ELs and ELw populations would show signs of introgression with Iberian lynxes derived from direct hybridization.

The results from demographic modeling of the two-population pairs follow similar patterns. While divergence times vary among models, they are broadly consistent across population pairs, with the best models for both the ILa-ELw and ILa-ELs pairs supporting either a recent split 150 kya with relatively little postspeciation gene flow, or instead a much deeper split (600-800 kya) followed by higher levels of gene flow. The migration rates themselves, on the other hand, differ considerably among population pairs, and are consistently higher between ILa and ELw than between ILa and ELs in the most recent times across all models. The Western EL population was already observed to have more signals of introgression with the Iberian lynx than the Eastern EL population in a previous study (Lucena-Perez et al. 2024). Our results indicate that the Western EL might also share more alleles with the Iberian lynx than the ELs population. Overall, by examining the differences in the amount of introgression detected in the different populations, it appears that, among the extant EL populations, the ELw is the one most closely related to the extinct EL population that hybridized with the Iberian lynx in Southwestern Europe since the late Pleistocene until very recently.

### Demographic history affects introgression

Demography, in terms of effective population size differences, can have significant effects on the amount of introgression occurring between pairs of populations, mainly because of how it interacts with mutational load and genetic drift (Whitlock et al. 2000; Harris and Nielsen 2016; Kim et al. 2018). Although the importance of neutral genetic diversity for the conservation of wild populations has been recently the subject of debate (DeWoody et al. 2021; Kyriazis et al. 2021; Teixeira and Huber 2021), inbred populations with low effective sizes might benefit comparatively more from the injection of genetic diversity from a divergent population (Ingvarsson and Whitlock 2000; Bierne et al. 2002). This is because the heterosis caused by the masking of genetic load accumulated in the inbred population might exceed the fitness loss caused by deleterious, incompatible, or maladaptive alleles entering the population via migration (Whitlock et al. 2000; Wang et al. 2017). Selective forces against mildly deleterious alleles entering the population through migration might also not be strong enough for selection to efficiently remove them if the receiving population size is too small (Moran et al. 2021), potentially leading to higher levels of observed introgression in smaller recipient populations. On the other hand, introgression from small populations may carry relatively more deleterious mutations, which would be more efficiently removed from large recipient populations (Juric et al. 2016), leading to an asymmetry in the observed amounts of introgression.

Although limited by the number of populations analyzed, our observations are partially consistent with these patterns, with the two lowest effective size populations, ILa and ELw, showing the highest amount of introgression in their genome. Previous work involving ELw and ELs populations showed that higher genetic diversity and lower inbreeding were observed in the Caucasian (ELs) population compared with other EL populations (Bazzicalupo et al. 2022). Our demographic modeling also shows that in all models the most recent population sizes of ELw are almost half of those of ELs, consistently following a downward trend in the last thousands of generations. In addition to the phylogeographic implications discussed above, the relatively higher effective population size of ELs might thus be another determining factor behind its lower observed introgression proportion. In contrast, the Iberian lynx population size has remained consistently smaller than that of both EL populations throughout their evolutionary history, both in our demographic models and previous reconstructions (Abascal et al. 2016; Godoy et al. 2025). The similar levels of introgression observed in ILa and ELw suggest that, although theoretically a smaller long-term effective population size should translate in higher rates of introgression due to weak selection against mildly deleterious alleles or heterosis due to the masking of fixed load, it does not appear to have had this effect in the Iberian lynx.

### Introgression as a source of genomic variation

Demographic history and migration are also expected to have important consequences for the levels of genetic diversity detected in populations. Our results are in line with the expectation that divergent alleles entering a recipient population through gene flow would introduce novel genetic variation and increase genetic diversity. The magnitude of this increase is relatively consistent across lynx populations, with introgression boosting local genetic diversity by approximately 3-fold in affected genomic windows. The slightly larger relative impact of introgression on diversity in ILa is also expected given the lower background levels of diversity in this population relative to ELw and ELs. This injection of diversity through migration, already observed by Lucena-Perez et al. (2024), might have represented an important offset to the loss of adaptive genetic diversity caused by small effective population sizes throughout the history of the ILa and ELw populations. In particular, we observe that genes that have experienced introgression from the EL to the Iberian lynx are enriched in functions related to the immune response, with many related to major histocompatibility complex (MHC) antigen interactions.

**Figure 5:**
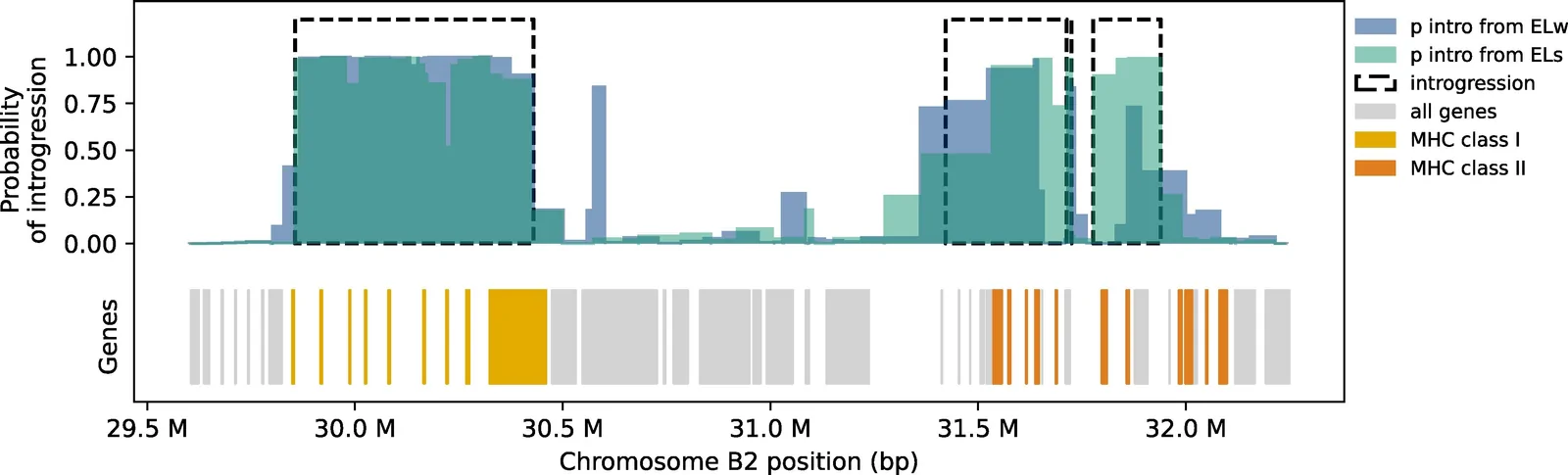
Introgression probability (top) from both Eurasian lynx populations (ELw and ELs) into the Iberian lynx (ILa) population along the genomic region occupied by MHC class I and class II genes (bottom). Introgressed windows are delimited by a black dashed box.

The MHC genes are organized in relatively large genomic clusters in several species, including humans, dogs, and cats (Yuhki et al. 2007). Within these clusters, genes with related antigen-presenting functions are spatially grouped and classified into distinct classes (eg Class I, II, and III) based on structural and functional characteristics. The annotated MHC class I and class II gene clusters, located near each other on chromosome B2, are both occupied by three distinct windows with signals of introgression from both EL populations into ILa (Fig. 5). Balancing selection has been observed to be a determining force in maintaining high levels of MHC polymorphisms in wild, highly eroded populations (Wenink et al. 1998; Aguilar et al. 2004; Wang et al. 2009; Wei et al. 2010), and was also hypothesized to be behind the high levels of MHC allele sharing among different lynx species (Marmesat et al. 2017). These genomic regions were also observed to maintain divergent haplotypes with distinct antigen-binding properties in the Iberian lynx (ie MHC supertypes; Marmesat et al. 2017), compared with the extreme levels of overall genomic erosion observed across the genome (Abascal et al. 2016). We note that since both balancing selection and introgression differ fundamentally in that the former pushes back cross-population coalescence times while the later makes them more recent, because both increase levels of interpopulation diversity it is possible that our classifier may produce an increased rate of false positives in regions experiencing balancing selection. A key factor in distinguishing between these two alternative hypotheses is the length of the shared haplotypes. Recent introgression events are expected to produce relatively long shared haplotypes between the two species, whereas ancestral polymorphisms maintained through ILS or balancing selection should appear in much shorter tracts, due to the cumulative effects of recombination over time (Gravel 2012; Liang and Nielsen 2014). Visual inspection of population alignments in the MHC regions reveals patterns of shared polymorphisms occurring sometimes in relatively long tracts—spanning in some cases almost the entire 128-SNP window analyzed by our deep CNN model (Figures S12–S15). While these results are not conclusive and remain open to interpretation, they seem to suggest that, in addition to long-term balancing selection, interspecific introgression from the EL may have contributed to the unexpectedly high MHC diversity observed in the Iberian lynx. Adaptive introgression has already been identified as one of the main mechanisms through which different vertebrate species have acquired novel variation and possibly recovered lost diversity in MHC genes (Gaczorek et al. 2024). Future studies should focus on testing this hypothesis in lynx populations more directly by seeking to jointly consider the impact of both balancing selection and introgression in the MHC region.

Although assisted gene flow between populations of different species is usually discouraged in conservation programs (Frankham et al. 2011; Aitken and Whitlock 2013), natural migration, even across species boundaries, probably played an important role in maintaining the genetic diversity of lynx populations through time. Conservation measures might allow higher flexibility with regards to source populations for assisted gene flow in circumstances similar to those faced recently by the Iberian lynx. When no additional conspecific populations exist, a recently diverged with evidence of recent or recurring admixture might represent an alternative natural reservoir of novel genetic variation. Still, caution and constant genetic monitoring are warranted to avoid the genomic extinction risks associated with outbreeding depression and genetic swamping (Todesco et al. 2016).

### Recombination and selection shape the introgression landscape

In general, the introgressed genome fraction in the receiving population can be highly variable, with multiple factors determining its amount. The two most prevalent are the number of generations since the gene flow event and its intensity, although these two factors alone are not always enough to explain the amount of introgression observed in natural populations (Edelman and Mallet 2021). One additional driver is the selection coefficient of the introgressed material, which interacts with genetic drift in governing allele frequency changes in the receiving population, favoring positively selected loci and eliminating the negatively selected ones. Recombination rate will then determine the rate at which neutral or beneficial introgressed alleles can be broken away from linked negatively selected loci entering the population with them (Martin et al. 2019). Higher recombination rates thus translate to higher tolerance to introgression, as purging of deleterious introgressed ancestry is more efficient in higher recombination regions (Veller et al. 2023). This effect is observable in chromosomal regions of different recombination rate (Schumer et al. 2018), but is also true at the genome level in species with higher or lower average recombination rates (Veller et al. 2023), and in small versus large chromosomes (Edelman et al. 2019), with the former generally having higher per base-pair recombination rates.

Our results are generally consistent with expectations with respect to recombination and selection on introgressed material: while we found introgressed windows on each chromosome in all three populations, introgression affected larger portions of smaller chromosomes and regions closer to the telomeres, both of which have higher recombination rates (Li et al. 2016b). Interestingly, the negative correlation between the fraction of windows with introgressed alleles and chromosome length (which should be inversely proportional to recombination rate) was strongest in the Iberian lynx. This may suggest that recessive deleterious alleles from donor populations were purged at a higher rate in this smaller recipient population, underscoring the potential deleterious consequences of gene flow in threatened populations (Kyriazis et al. 2021). On the other hand, we found similar amounts of gene sequences in the introgressed windows of the three lynx populations as in random subsamples of the genomes. These latter results may suggest that selection in the receiving populations did not strongly favor the elimination of gene sequences coming from the other species, and we note that these genes were enriched in an array of different biological functions. More precise and detailed inferences regarding variation in the strength of selection and the recombination rate along the genome might help clarify how the interaction between these two factors has shaped the genomic introgression landscape of lynxes.

### Summary and conclusions

In this study, we applied a deep learning approach to quantify and, for the first time, localize regions with signals of interspecific introgression in the genomes of three lynx populations. This framework allowed us to measure the proportion of the genome with signals of introgression in each population, identify which EL clade is the most likely source of introgression in the Iberian lynx and describe the long-term consequences of introgression on the genomes of these species.

By comparing the extent of introgressed genomic regions across two EL populations, we found evidence suggesting that the Western EL clade is the closest extant lineage to the extinct Eurasian population that served as the source of the observed introgression. Our results are consistent with theoretical expectations that introgression should be more prevalent in populations with lower effective population sizes, such as the ELw and the Iberian lynx, and in genomic regions with higher recombination rates, including smaller chromosomes and regions closer to telomeres. Two observations did however deviate from theoretical expectations. First, despite its historically lower effective population size, the Iberian lynx does not show higher overall levels of introgression compared with the more abundant ELw. Instead, the Iberian lynx population shows evidence suggestive of stronger selection against deleterious introgressed alleles than observed in the other populations (Fig. 3). Second, despite being theoretically subject to relatively higher levels of purifying selection, coding sequences appear to be just as likely to introgress as intergenic regions. A possible speculation is that donor populations harbor more recessive deleterious alleles in noncoding regions than in coding regions, thereby somewhat offsetting the expected stronger selection against individual deleterious mutations in coding regions.

Finally, we detected an approximately 3-fold increase in genetic diversity associated with introgression across populations, regardless of their demographic histories. Although limited in the number of observations, this somewhat consistent effect provides a measurable benchmark for predicting the long-term consequences of gene flow on the genetic diversity of populations. In the case of the Iberian lynx, our results suggest that the negative effects of natural gene flow from the closely related EL were at least partially offset by the benefits of an increased genetic diversity, like increasing the number of available MHC haplotypes. Although the necessary caution is still warranted and advised, these findings support the idea that assisted gene flow could in some cases represent a viable conservation strategy in extreme situations, such as those faced by the Iberian lynx prior to its recent conservation-driven recovery.

## Methods

### Sampling, DNA extraction, and sequencing

In this study we generated new resequencing data for 10 individuals of Iberian lynx, which we analyzed alongside already published whole genome sequences from 44 individuals of both Iberian and EL (Bazzicalupo et al. 2022; Kleinman-Ruiz et al. 2022; Table S1). DNA was extracted from tissue and blood samples using a combination of silica-coated paramagnetic beads (NucleoMag® Tissue, MACHEREY-NAGEL GmbH & Co. KG) and a classical phenol–chloroform protocol in LEM-EBD facilities (Seville, Spain). gDNA samples were then sent for paired-end library preparation and sequencing on Illumina NovaSeq X Plus platform at Novogene Ltd facilities (Cambridge, UK). Table S1 lists details regarding each sample's origin, original sequencing study, sequencing technology used and average sequencing depth. In all cases, primary data analysis was carried out with the standard Illumina pipeline. Sequencing reads were processed using the software fastp (Chen et al. 2018) and quality control of processed reads was carried out using the software fastqc (https://www.bioinformatics.babraham.ac.uk/projects/fastqc). We enabled base correction using overlapping paired sequences, trimmed adapters and poly-G sequences and selected a minimum length of 30 base pairs per read after trimming.

### Sequencing reads alignment, variant calling, and initial filtering

To avoid reference bias in our analyses, sequencing reads were aligned to a recently generated *Lynx rufus* reference genome (mLynRuf2.2, https://denovo.cnag.cat/lynx_rufus_data), using BWA-MEM (Li 2013). After alignment, duplicate reads were marked with the MarkDuplicates function of the software package Picard Tools (http://broadinstitute.github.io/picard), and INDELs were realigned using GATK RealignerTargetCreator and IndelRealigner (McKenna et al. 2010). We used the software Qualimap 2 (Okonechnikov et al. 2016) to assess the alignment quality and calculate average sequencing depth for each sample (Table S1).

Variant detection and calling were performed using the software GATK HaplotypeCaller v4.2 (McKenna et al. 2010), which generated a genome variant calling format (GVCF) file for each of our individuals. These were then combined into a single GVCF of all of the individuals using the CombineGVCFs tool of GATK. Finally, the GenotypeGVCFs tool was used to call variants and genotypes of all of the individuals in the unified GVCF. We limited our analysis to the scaffolds of the reference genome assigned to one of the 18 autosomes.

We identified low-complexity regions and interspersed repeats in the reference genome using the combination of RepeatModeler and RepeatMasker softwares (http://www.repeatmasker.org). Any variant found within these regions, was removed from the VCF together with any INDEL and nonbiallelic variant. Low quality variants were also filtered out following GATK's suggested thresholds of variant quality (ie FS ≥ 60, MQ ≤ 40), using a slightly stricter threshold for the quality by depth parameter (QD < 6 instead of the suggested QD < 2), after inspecting the genome-wide distribution of values of these statistics.

We then separated our dataset into three distinct VCFs, one for each population, to perform population specific variant filtering. From each of the three population VCFs, we filtered out any SNP with 15% or more missing data. Additionally, to avoid regions with multiple paralogs collapsed in the reference genome, mean read depth in consecutive 10 kbp windows along the genome of each individual was calculated using the software Mosdepth (Pedersen and Quinlan 2018). For each window we then calculated the sum of sequencing depth in each population and excluded any window with a total sequencing depth higher than 1.5 times the mode. We joined the filtered Iberian lynx VCF with each of the EL filtered VCFs, creating two distinct population-pair datasets for downstream analysis: the ILa-ELw pair and the ILa-ELs pair.

### Demographic inference

To train a classifier to detect introgression, we first had to reconstruct the demographic history of each population pair to be used during training simulations. In order to reconstruct the joint demographic history of the Iberian and EL population pairs, we ran the software GADMA2 (Noskova et al. 2023). GADMA2 has the advantage of providing an option for dynamic demographic model construction through its *structure model* specification. This provides a more data-driven and unsupervised approach to demographic modeling by using genetic algorithms for global and local optimization (Momigliano et al. 2021), to dynamically explore the likelihood space under different numbers of epochs before and after population splits, as well as different population size change functions. We further filtered our dataset for running GADMA2 in optimal conditions. Specifically, to reduce the bias introduced by selection on demographic inference, we used bedtools (Quinlan and Hall 2010) to remove any SNP found within annotated genes. Additionally, we pruned the resulting SNP dataset to reduce the amount of linkage disequilibrium between SNPs and to remove missing data. The latter step allowed direct likelihood comparison between model runs using the full SFS. We did so by using the software vcftools (Danecek et al. 2011), with –thin 50,000 to remove SNPs at physical distances of <50,000 bp between each other and –max-missing 1 to remove any SNP with missing data.

We ran GADMA2 in *structure model* mode, specifying a final model structure with two epochs before and three after the population split, using the length of the different time spans, the effective population sizes, the growth rates of the active populations, and the migration rates among them as model parameters (Figure S16). We set a minimum of 20,000 generations for the divergence time between lynx species, allowing the population size changes to either be exponential or instantaneous. The default parameter bounds were changed to be [0.001–50] and [0–10] times the ancestral population size for each effective population size and event time, respectively. We selected *moments* as the engine for frequency spectrum (FS) simulation and model evaluation, and set the per base per generation mutation rate to 6e-9 (Abascal et al. 2016). Following the suggestions included in Noskova et al. (2023), we selected a hyperparameter configuration for the genetic algorithm that best performed with the *moments* engine: Size of generation = 10, N elitism = 3, P mutation = 0.2, P crossover = 0.3, P random = 0.2, Mean mutation strength = 0.776, Const for mutation strength = 1.302, Mean mutation rate = 0.273, and Const for mutation rate = 1.475. With the intention of thoroughly exploring the likelihood landscape, a total of 500 distinct runs of the genetic algorithm were performed. Finally, we compared the likelihoods of the best model of each of the 500 runs, selecting the highest scoring models. Local optimization was then performed on the best scoring models, using 100 block bootstrap replicates, with a chunk size of 200 kbp, of the dataset including all of the SNPs before pruning. The local optimization algorithm was run using GADMA's *run_ls_on_boot_data* script, which implements *dadi*'s optimize_log function, and consists in using the BFGS method to optimize log-transformed parameter values. Median and 95% confidence intervals of each parameter were calculated from the optimized bootstrap replicates.

### Phasing variants

In order to perform genome scans for introgression, we needed phased haplotype data, which we obtained from genotype data of each lynx population. We first split our data into three distinct VCFs, one for each of the three populations, and then applied a two-step approach. We first used WhatsHap v1.1 (Martin et al. 2016) to obtain phase sets (−tag = PS) from individual reads and population data. Next, we used SHAPEIT v.4.2.1 (Delaneau et al. 2019) to infer each sample's haplotype from the phase set data. An expected error rate of 0.0001 and “10b,1p,1b,1p,1b,1p,1b,1p,10m” MCMC iterations were used as suggested by the SHAPEIT4 manual. Once phased, the variants from each EL population were joined with the ILa variants, creating two-population pair phased datasets for the introgression scans (phased ILa-ELw and phased ILa-ELs). As the phasing process also imputes any missing genotypes in the phased samples, the resulting phased datasets have no missing data.

### Introgression scans

#### Methodological approach

To identify genomic windows with signals of introgression in either direction between population pairs, we used the introNets package (https://github.com/SchriderLab/introNets; Ray et al. 2024) to implement a discriminator model trained to look at haplotype SNP data for a particular genomic window and distinguish between different introgression scenarios. The discriminator model is a Residual Network (ResNet34), a type of deep CNN architecture that was developed for general image classification tasks (Koonce 2021), and was recently implemented to detect introgressed regions between closely related species with high accuracy (Ray et al. 2024). Other than the shape of input tensors (see below), the original ResNet-34 architecture was used without modification (He et al. 2015). It consists of an initial convolutional block (7 × 7 convolution, batch normalization, ReLU activation, and max pooling), followed by 16 basic residual blocks (32 convolutional layers) arranged in four stages, with identity or projection skip connections within each residual block. The network terminates with global average pooling and a fully connected classification layer.

In order to format the genomic windows for the model to classify, we used genotypes observed in phased biallelic SNPs to generate three dimensional tensors (*l* × *n* × *m*), which would be passed as input to the CNN the same way the original ResNet34 model handles multilayered images. The first dimension of the tensor (*l*) is the number of populations in the alignment, the second dimension (*n*) is the number of phased haplotypes observed in each population (two for each sequenced diploid individual), and the third dimension (*m*) is the number of SNPs in the window. As we are analyzing introgression between pairs of populations, the first dimension of the tensor is always equal to 2. Although the number of observed phased haplotypes changed in each population (ILa: 44, ELw: 40, and ELs: 24), the tensor needs to have the same number of haplotypes in both populations for the image to be correctly formatted. To solve this issue and to not lose information on any of the sequenced haplotypes, we selected the highest number of observed haplotypes in any population (44) as the second dimension and upsampled the populations with fewer haplotypes, selecting observed haplotypes at random to appear multiple times in the input tensor (following Ray et al. 2024). The third dimension was set to 128, which is the number of SNPs included in each genomic window we analyzed. This resulted in tensors of shape (2, 44, and 128) for all pairs of populations analyzed. The value of a particular entry in the tensor is the genotype observed at a particular SNP, in a particular haplotype, in a particular population. Since the SNPs are biallelic, the resulting tensors have binary values in their entries, 0 for the major allele and 1 for the minor one. The discriminator model was trained to look at a particular tensor representing genotypes of a genomic window and assign a probability to each of 4 distinct scenarios that might have generated the resulting alignment: “ELtoIL” where introgression from an EL population is observed in the Iberian lynx population; “ILtoEL” where introgression from the Iberian lynx is observed in an EL population; “BiDir” where we observe introgression from the other population in both an EL population and the Iberian lynx; and “none” when no introgression is observed.

#### Simulation and training

To train the model to perform the discrimination task, we ran coalescent simulations under each of the three selected demographic models of each population pair for each introgression scenario, using a modified version of the software ms (Hudson 2002) called msmodified, developed by Durvasula and Sankararaman (2019) and available from the ArchIE (https://github.com/sriramlab/ArchIE) and introNets packages. When generating data for the cases without introgression (“none” scenario) the demographic history was left unmodified. When simulating cases with introgression we added to the demographic model a mass migration event in the appropriate direction, from the EL population to the Iberian lynx for “ELtoIL” cases, from the Iberian lynx to the EL population for “ILtoEL” cases and in both directions for “BiDir” cases. The mass migration event was simulated by moving a proportion of lineages from one population into the other at a given time. The proportion was randomly selected to be between 5% and 50% of the source population lineages, while the timing of the event was randomly selected between 1 and 5,000 generations before present. Tensors of the appropriate shape (see above) were generated from the simulated data, making sure, based on the required introgression case, that introgressed haplotypes were present in the receiving population; this information is recorded by msmodified. For each population pair, the final set of simulations for training consisted of 12,000 simulations of each introgression scenario, simulated under each of the demographic models reconstructed for the population pair, for a total of 144,000 training tensors. The total simulated dataset was split into training and validation sets, with the latter comprising 5% of the simulations for each introgression scenario. Model training was performed from scratch using the categorical cross entropy as loss function, the Adam optimizer with default settings (learning rate = 0.001 and β_1_, β_2_ = 0.9, 0.999; Kingma and Ba, 2014), as described in Ray et al. (2024). We used a batch size of 16 and maximum training time was set to 100 epochs, with 1,500 training steps in each epoch, and a maximum of 10 consecutive epochs without validation loss decreasing before early termination of training. Throughout training, the set of weights that produced the lowest validation loss up to that point was recorded, and upon termination these weights were used as the final classification model. Two distinct models were trained, one for each population pair.

#### Model performance

The performance of the model was evaluated using 1,000 additional simulations under each introgression scenario with each demographic model. The model's output was transformed into a probability score for each introgression scenario using the softmax function. During model evaluation, we assigned a particular window to a particular introgression category using the following criteria: if the sum of the probabilities of “ELtoIL” and “BiDir” was higher than a probability threshold *P*, we assigned the window to the “ELtoIL” category; if the sum of the probabilities for “ILtoEL” and “BiDir” was higher than *P* then the window would be assigned to “ILtoEL”; if a window is assigned to both “ELtoIL” and “ILtoEL,” then its prediction is changed to “BiDir”; if the probability threshold was not exceeded for any of these cases, then the window was not assigned to any introgression category and was instead categorized as “none.” We use the sum of single-direction and bidirectional gene flow to account for cases where the individual probabilities of each scenario are individually low, but together they represent evidence that introgression in at least one direction has happened.

We evaluated model performance using five increasing values of *P* (0.75, 0.8, 0.85, 0.9, and 0.95), comparing model precision and recall in detecting presence or absence of introgression in a given genomic window, and also in correctly identifying introgression directionality in windows where introgression was correctly identified. Model precision is defined as the proportion of correctly called cases (true positives) to the total amount of cases called as a particular scenario (true positives + false positives). Recall on the other hand is the proportion of true positives to the total number of cases simulated under a particular scenario (true positives + false negatives). Based on these evaluation results, we sought to choose a value of *P* that achieved high precision while calling introgression, and also keeping acceptable levels of recall.

Once the optimal *P* was selected, we further evaluated model performance by simulating and analyzing additional evaluation datasets. First, to understand the effects of low sequencing depth on model inference, we generated evaluation datasets of average sequencing depth ranging from 2× to 6×, emulating wrong homozygote calls caused by allelic drop-out at heterozygous positions at sites with low number of sequencing reads. At each heterozygous position, in all simulated individuals, the number of sequencing reads was sampled from a Poisson distribution with mean equal to the average depth for that particular evaluation set. A minimum depth of one read was enforced to reflect the absence of missing data in our real data application (see “Phasing variants”). Given the sampled depth, reads were assigned to reference and alternative alleles using a binomial sampling process with equal probability (*P* = 0.5) for each allele. Genotypes were then called based on the observed allele counts: sites with only reference reads were called homozygous reference, sites with only alternative reads were called homozygous alternative, and sites with both were called heterozygous. This procedure is aimed at simulating stochastic read sampling and genotype calling error due to finite sequencing depth at heterozygous sites.

Additionally, we built a “realistic” evaluation set that included both the presence of low-depth individuals and incorporated heterogeneity in mutation and recombination rates. For each population, a number of individuals equal to the number of low sequencing samples in our dataset (12 for ILa, 12 for ELw, 1 for ELs; see Table S1) was marked as low-depth. We then introduced genotyping errors at heterozygous positions in these low-depth individuals as described above, assuming an average read depth of 4×, a conservative value given that all of our low-depth individuals have at least 5× average depth. To incorporate heterogeneity in mutation and recombination rates across genomic regions, in each simulation values were sampled from a gamma distribution centered on the genome-wide average. For each simulated region, a scaling factor was drawn from a gamma distribution with shape parameter *α* = 10 and scale parameter 1/*α*, resulting in a gamma distribution with mean 1. The rates were then obtained by multiplying the genome-averages by this scaling factor. This approach preserves the overall mean rates while introducing moderate among-region heterogeneity, allowing the evaluation of the effect on model performance of occasional hot- or cold-spots of mutation and recombination.

#### Introgressed windows and downstream analyses

Finally, the model trained for each of the two-population pair datasets was run on phased data from the corresponding dataset. Overlapping sliding windows along the genome, with a window length of 128 SNPs and a step size of 64 SNPs were transformed into input tensors of the desired shape, generating a total of 79,184 ILa-ELw genomic windows and 77,565 ILa-ELs genomic windows for the introgression scan. We assigned genomic windows to different introgression categories using a probability threshold (*P*) selected during model evaluation as discussed above. Consecutive windows assigned to the same introgression class were then merged, creating four sets of introgressed regions: (i) windows of introgression from ILa to ELw; (ii) windows of introgression from ILa to ELs; (iii) windows of introgression from ELw to ILa; (iv) windows of introgression from ELs to ILa. Additionally, the final dataset of genomic windows of introgression from EL into ILa was generated by merging introgressed windows from ELs and ELw into ILa.

To assess if the simulations used to train the model properly resemble real variation in lynx populations, we compared the SFS of observed genomic windows to the SFS of the genomic simulations used to train the classifier. For each population pair, we extracted the observed SFS from windows with signals of introgression in each direction and from windows without introgression. The simulated SFS were extracted from the concatenation of the training msmodified output files. Observed and simulated SFS were visually compared, and log-likelihoods for each comparison were computed using the *plot_2d_comp_multinom* and *ll_multinom* functions implemented in the ∂a∂i software package (Gutenkunst et al. 2009).

To explore which biological functions were associated with introgressed regions, we checked for functional enrichment in the BP Gene Ontology (GO) annotations of genes found in introgressed windows of the different populations. We used the R package “topGO” (Alexa and Rahnenfuhrer 2017) to perform statistical over-representation tests, comparing expected and observed number of occurrences of a given BP GO term, given the total number present in the reference genome. A *P*-value threshold of 0.01 was selected for the results of Fisher's exact test using the *weight01* algorithm.

We compared measures of genetic diversity, chromosomal position and gene content in introgressed windows in each of the recipient populations (ILa, ELw, and ELs) and compared them to a null model. The null model for a given population was composed of 100 datasets of windows of the same length as the introgressed ones, but placed at random locations along the genome obtained with bedtools (Quinlan and Hall 2010). We then measured nucleotide diversity (π), distance to the closest telomere, and base-pair overlap with annotated genes in both the introgressed and the random dataset and compared the distributions of these values in introgressed versus random windows. To test the statistical significance and the magnitude of the observed differences, two-tailed *t*-tests were performed on log-transformed data to obtain normally distributed values. Corresponding *P*-values and effect sizes –quantified as Cd– were obtained using the R package lsr (Navarro 2015).

## Supplementary Material

msag086_Supplementary_Data

## Data Availability

Sequences analyzed in this study are available on the European Nucleotide Archive under the primary accession numbers: PRJEB108892 (current study), PRJEB48088 (Bazzicalupo et al. 2022), PRJEB28038 (Lucena-Perez et al. 2020), and PRJEB44874 (Kleinman-Ruiz et al. 2022). The code generated to run all of the analyses included in the article can be found at: https://github.com/Enricobazzi/Lynxtrogression_v2
