# Correction to: Deep learning reveals genomic regions introgressed between two recurrently hybridizing lynx species

**DOI:** 10.1093/molbev/msag234

**Published:** 2026-09-15

This is a correction to: Enrico Bazzicalupo, Lorena Lorenzo-Fernández, Lucía Mayor-Fidalgo, Laura Soriano, Daniel R Schrider, José A Godoy, Deep learning reveals genomic regions introgressed between two recurrently hybridizing lynx species, *Molecular Biology and Evolution*, Volume 43, Issue 4, April 2026, msag086, https://doi.org/10.1093/molbev/msag086

In the originally published version of this manuscript, a funding source was omitted.

The following declaration has been added to the end of the ‘Funding’ section:

“D.R.S. received support from the National Institute of General Medical Sciences of the National Institutes of Health under award number R35GM138286”.

