# Supplementary material for: Deep learning reveals genomic regions introgressed between two recurrently hybridizing lynx species
Source: Mol Biol Evol. 2026 Apr 8;43(4):msag086. doi: 10.1093/molbev/msag086 (PMC13102358; doi:10.1093/molbev/msag086)
Supplement: msag086_Supplementary_Data [file msag086_supplementary_data.docx]

# **DEEP LEARNING REVEALS GENOMIC REGIONS INTROGRESSED BETWEEN TWO RECURRENTLY HYBRIDIZING LYNX SPECIES**

Enrico Bazzicalupo^1^, Lorena Lorenzo–Fernández^1^, Lucía Mayor–Fidalgo^1^, Laura Soriano^1^,

Daniel R. Schrider^2*^, José Antonio Godoy^1*^

**Supplementary Figures and Tables**


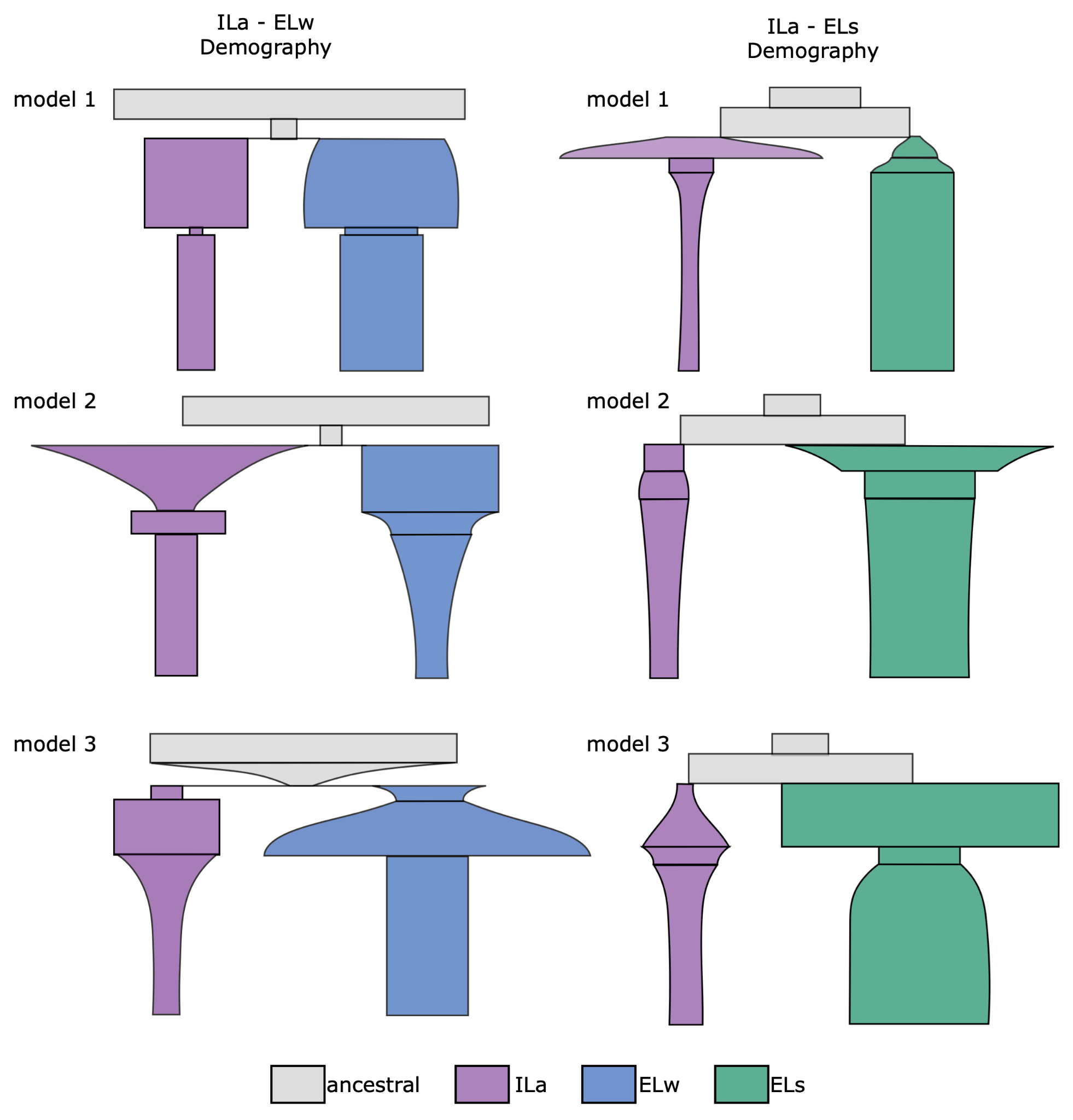


**Figure S1.** Tube-shape representation of the best three scoring demographic models for each population pair (ILa-ELw left; ILa-ELs right). Time is represented from top (more ancient) to bottom (more recent), with each epoch in each population represented by a polygon. Effective population sizes are proportional to width of polygons and are either constant or changing exponentially within an epoch.


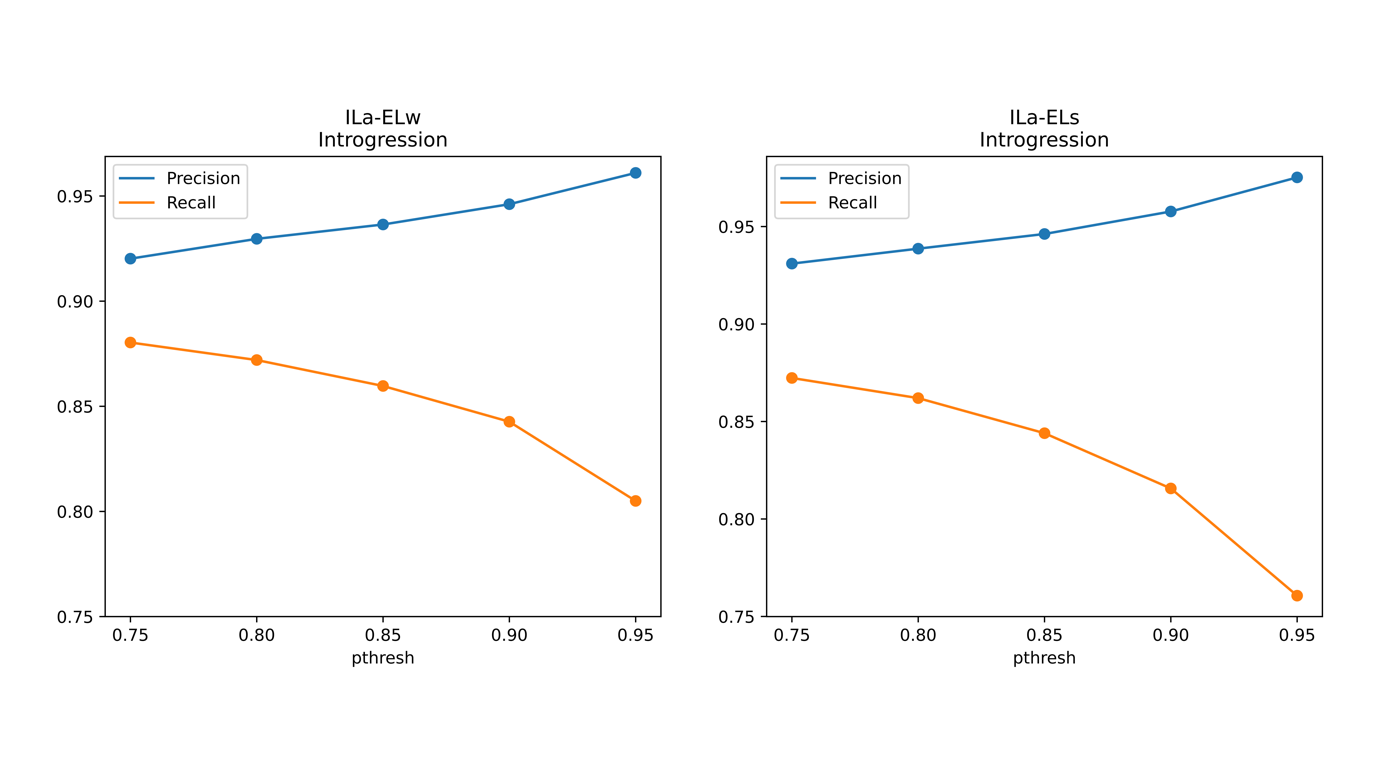


**Figure S2.** Precision and recall calculated across different probability thresholds (*p*) for assigning windows as introgressed, in models trained for the ILa–ELw dataset (left) and the ILa–ELs dataset (right).

**
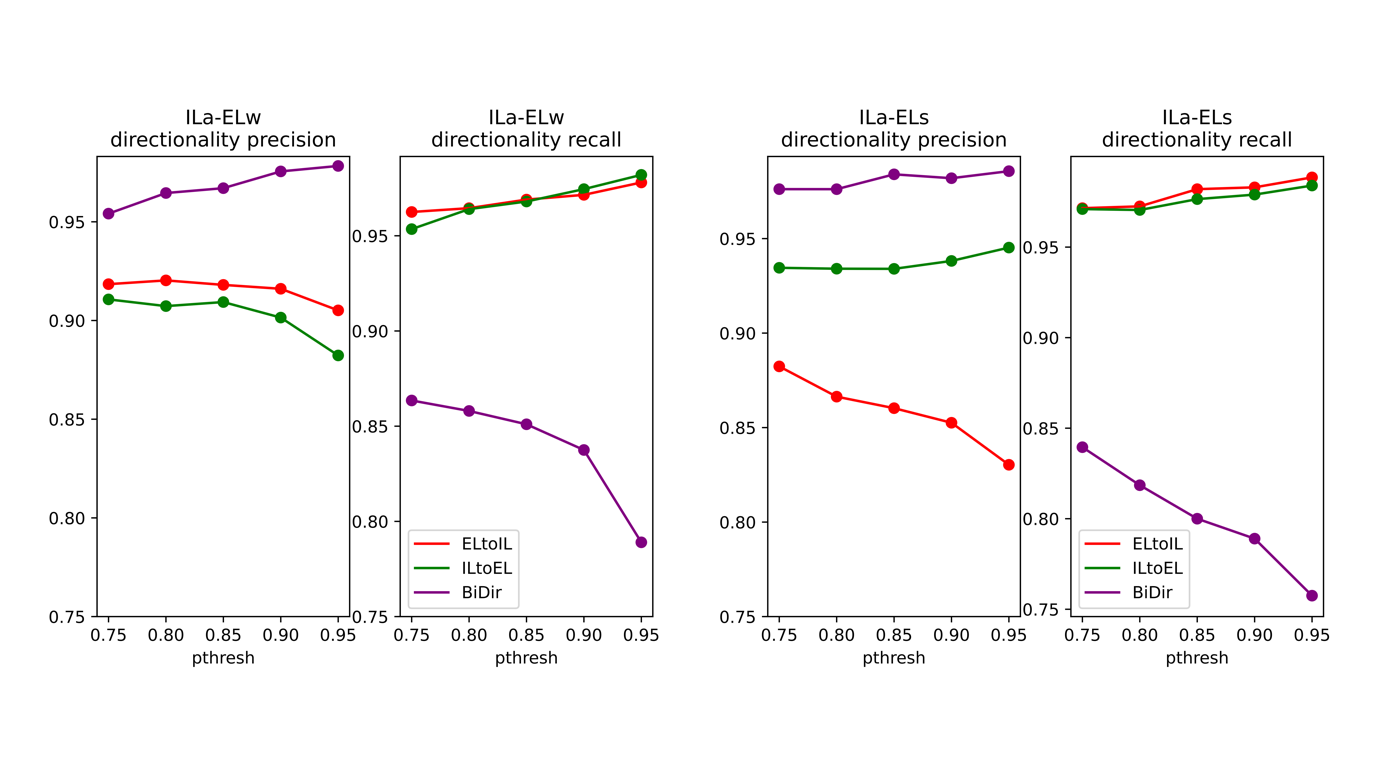
Figure S3.** Precision and recall calculated across different probability thresholds (*p*) for the directionality of introgression of windows correctly identified as introgressed, in models trained for the ILa–ELw dataset (left) and the ILa–ELs dataset (right).

**
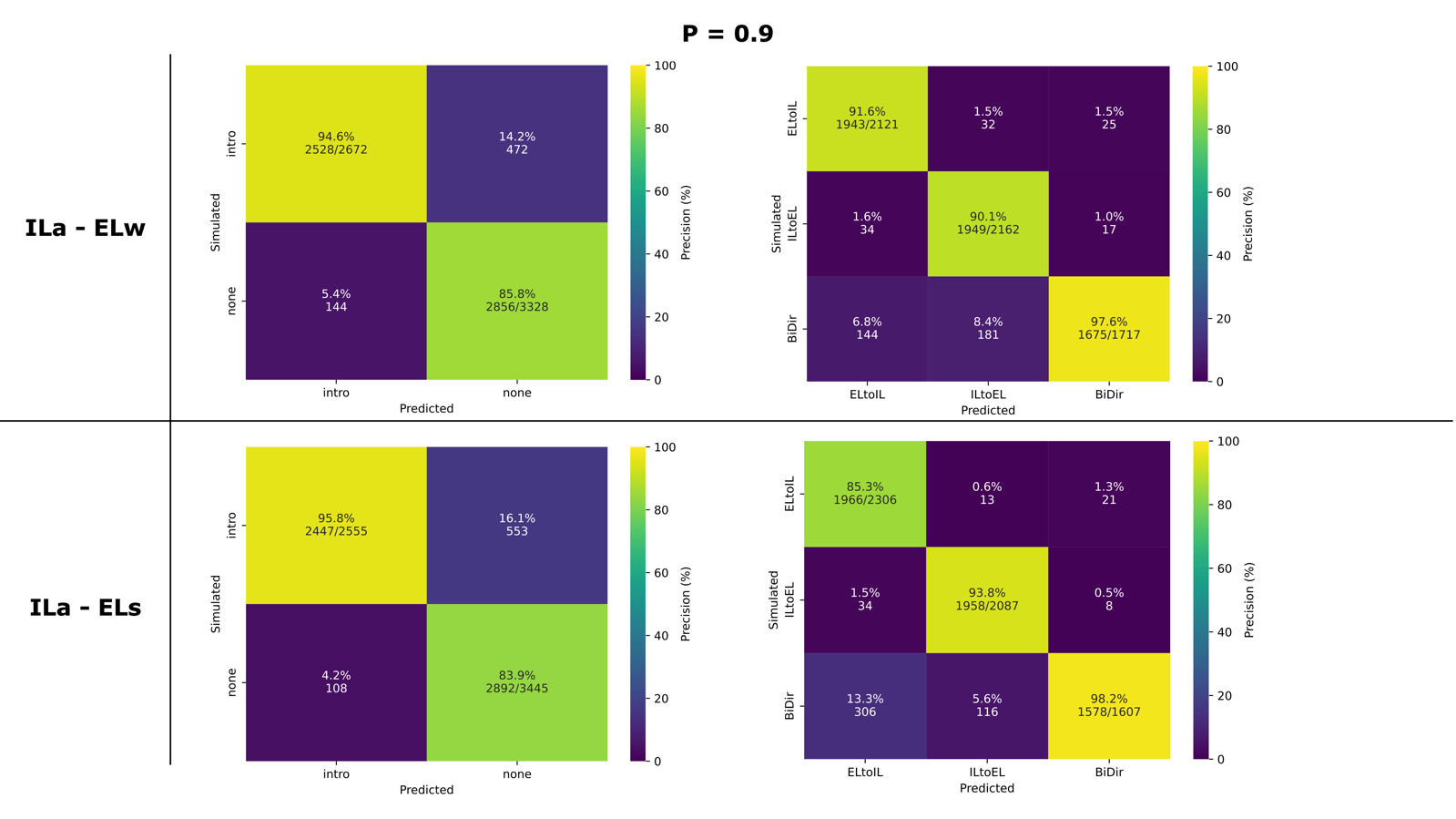
**

**Figure S4.** Confusion matrices obtained when selecting a probability threshold (*p*) of 0.9 for assigning windows as introgressed (left) and the direction of introgression of introgressed windows (right) in models trained for the ILa–ELw dataset (top) and the ILa–ELs dataset (bottom). Cells are color scaled based on Simulated / Predicted ratio (precision for true class on the matrix diagonal).

**
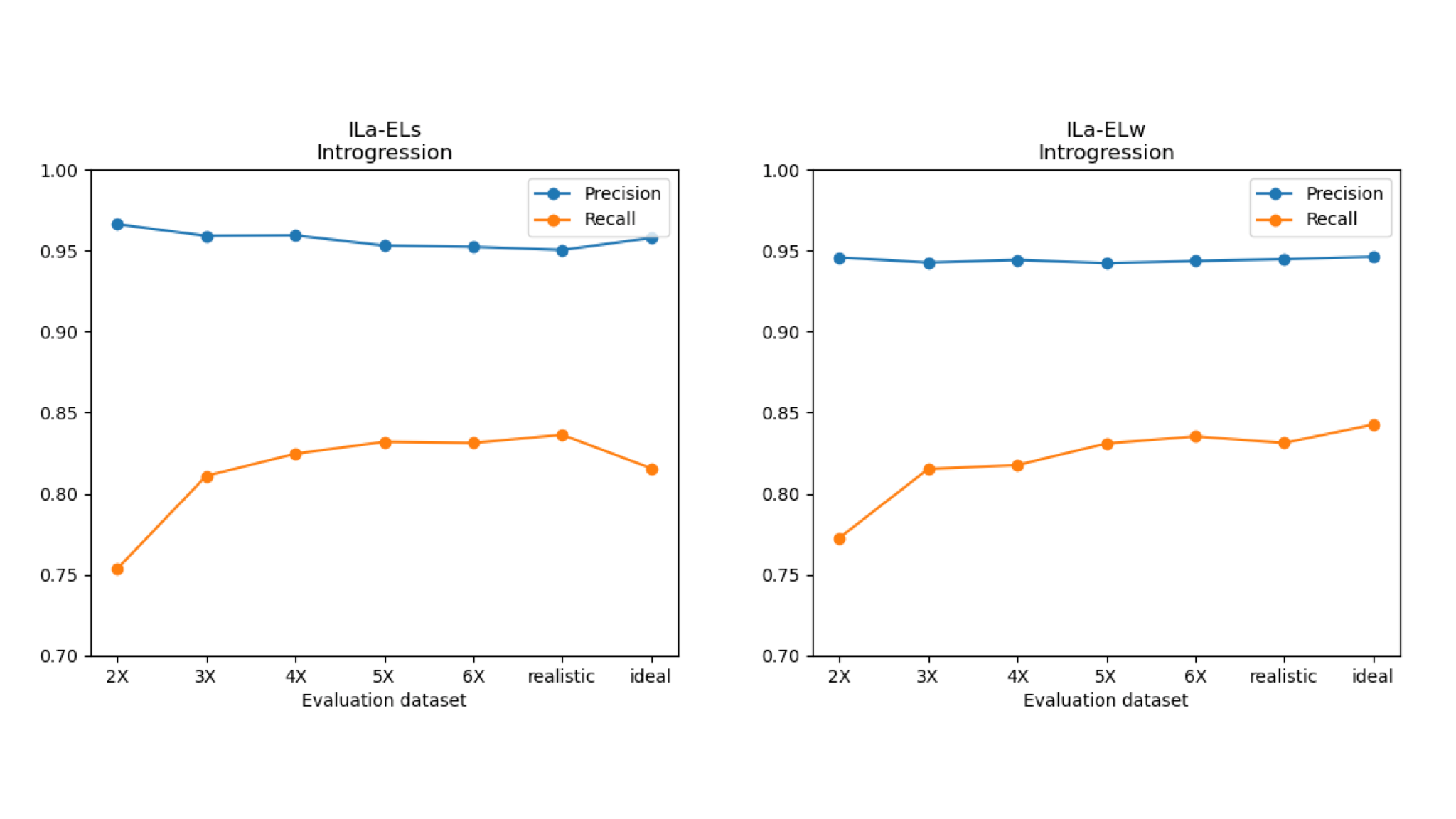
Figure S5.** Precision and recall in the assignment of windows as introgressed calculated in models trained for the ILa–ELw dataset (left) and the ILa–ELs dataset (right) using evaluation datasets generated to mimic varying sequence depths (2X-6X) or by considering both the presence of some low-depth individuals and variance in mutation and recombination rates (*realistic*), compared to “*ideal*” datasets (all individuals at high coverage and constant mutation and recombination rates).


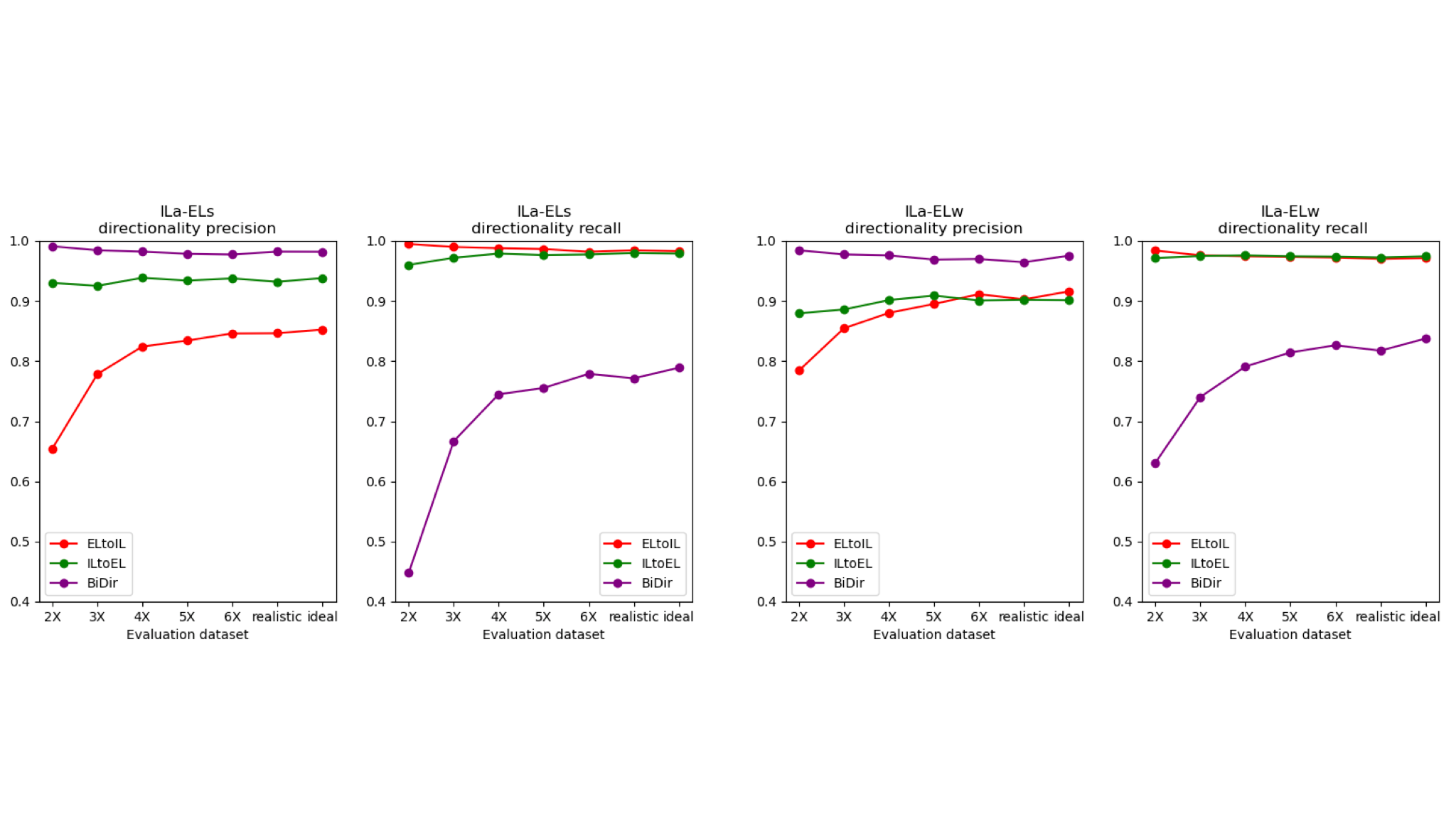


**Figure S6.** Precision and recall for the directionality of introgression of windows correctly identified as introgressed, in models trained for the ILa–ELw dataset (left) and the ILa–ELs dataset (right), calculated using evaluation datasets generated to mimic varying sequence depths (2X-6X) or by considering both the presence of some low-depth individuals and variance in mutation and recombination rates (*realistic*), compared to “*ideal*” datasets (all individuals at high coverage and constant mutation and recombination rates).


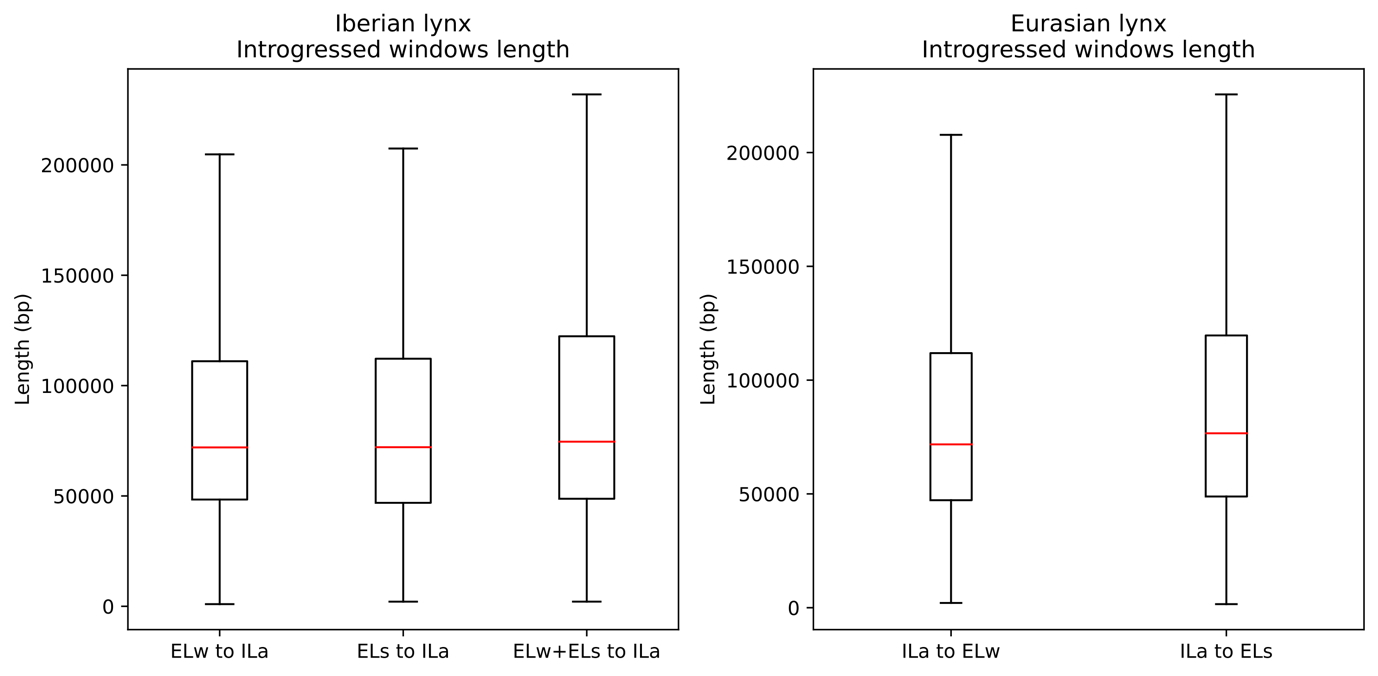


**Figure S7.** Boxplots of introgressed window lengths into the Iberian lynx from the two Eurasian lynx populations (left) and into the two Eurasian lynx populations from the Iberian lynx (right). Red lines represent median values, while 25th and 75th percentiles are represented by bottom and top whiskers, respectively.


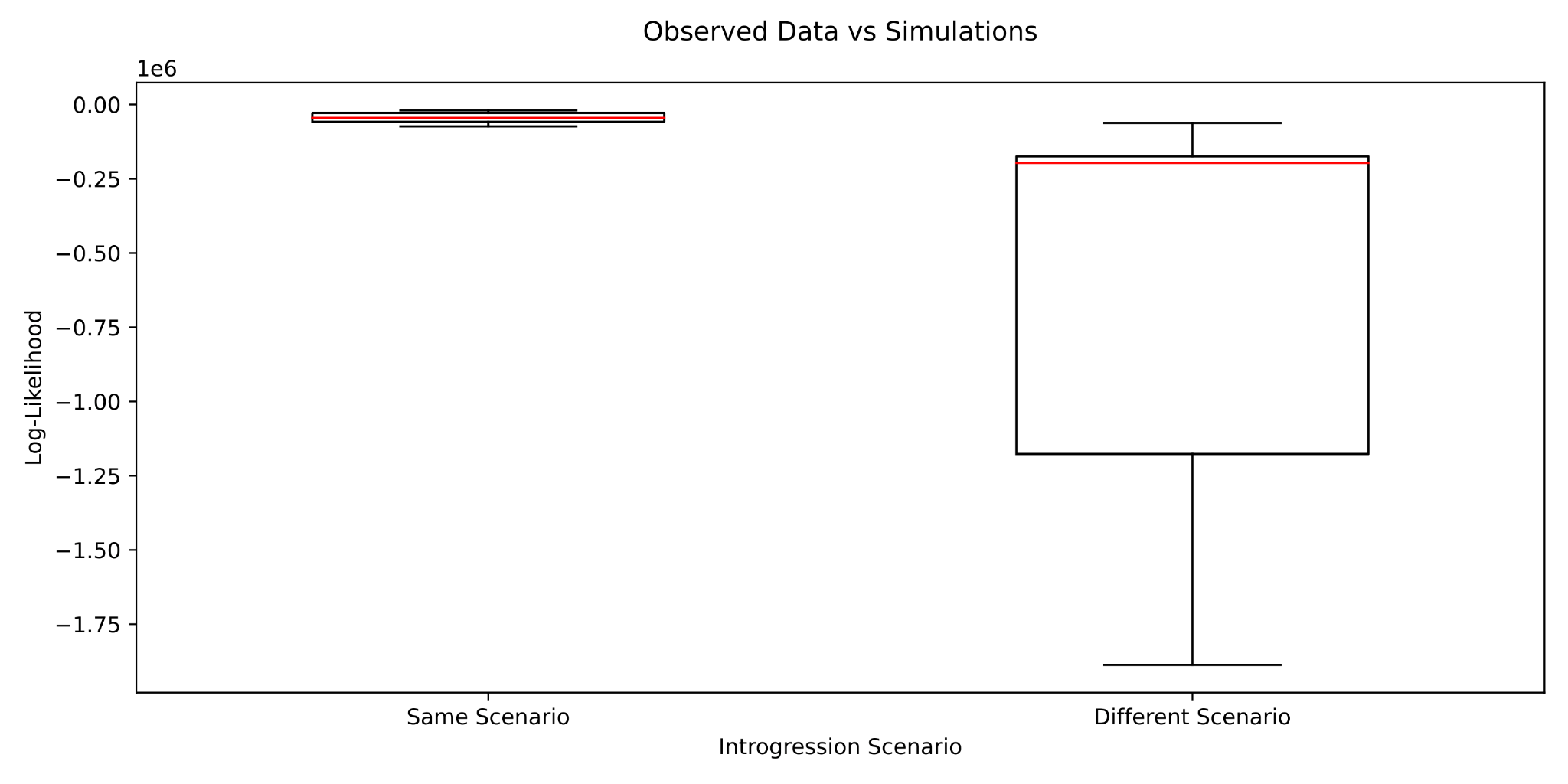


**Figure S8.** Boxplots of likelihoods of comparing observed genomic windows of a particular introgression scenario to training simulations of the same or different introgression scenario. Likelihoods are calculated from site frequency spectra comparisons run using the *ll_multinom* function implemented in the ∂a∂i software package.


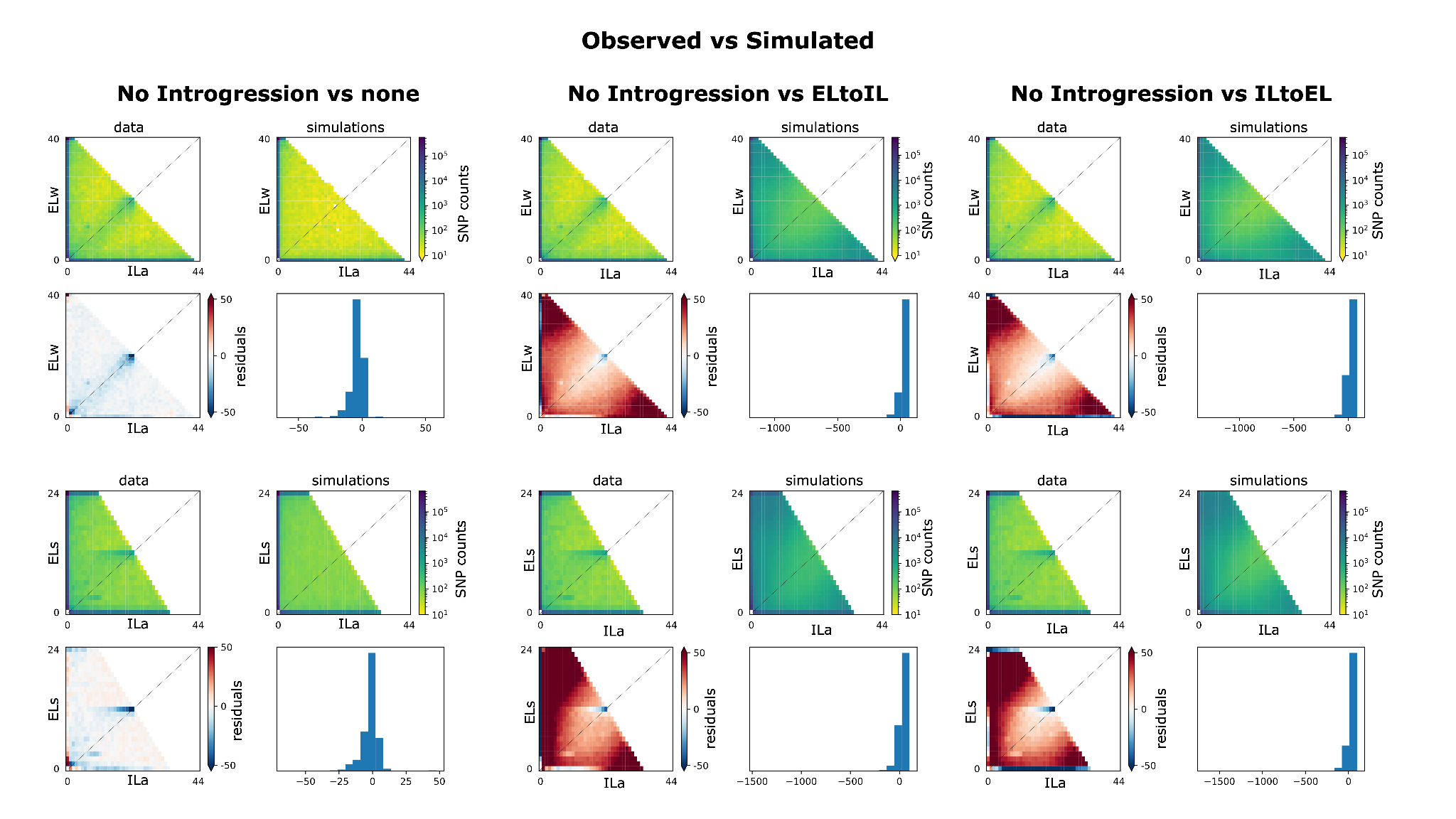


**Figure S9.** Two dimensional SFS comparisons between observed data with no introgression and training simulations run without pulse migrations (left), with ELtoIL introgression (center), and with ILtoEL introgression (right), in ILa-ELw (top) and ILa-ELw (bottom) models. Within each population pair’s panel, overall SNP counts are displayed above and Anscombe residuals from SFS cell comparisons together with overall residual distributions are displayed below. Red values indicate higher SNP counts in simulations, whereas blue values indicate higher SNP counts in real data.


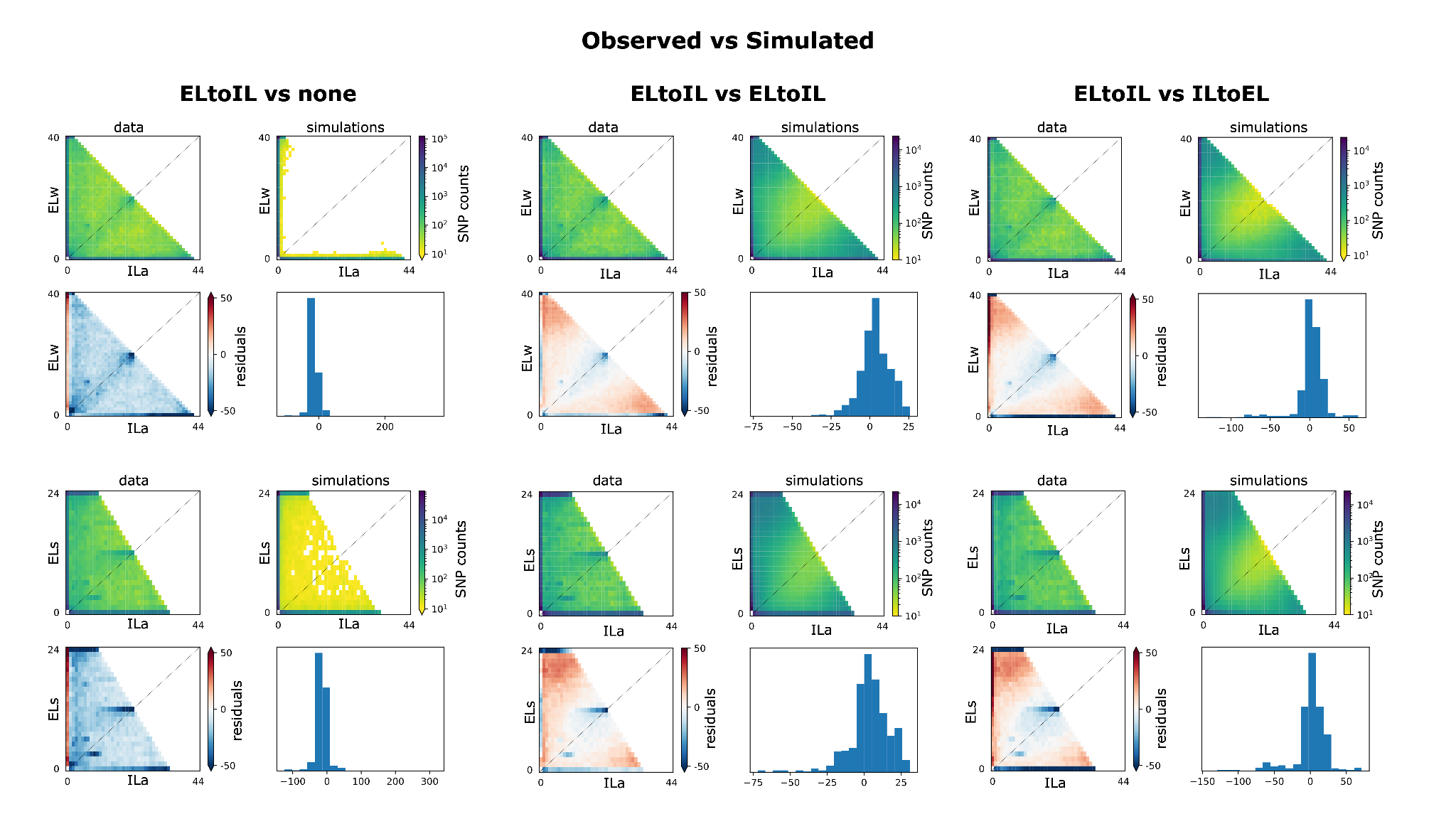


**Figure S10.** Two dimensional SFS comparisons between observed data with introgression into the Iberian lynx population and training simulations run without pulse migrations (left), with ELtoIL introgression (center), and with ILtoEL introgression (right), in ILa-ELw (top) and ILa-ELw (bottom) models. Within each population pair’s panel, overall SNP counts are displayed above and Anscombe residuals from SFS cell comparisons together with overall residual distributions are displayed below. Red values indicate higher SNP counts in simulations, whereas blue values indicate higher SNP counts in real data.

**
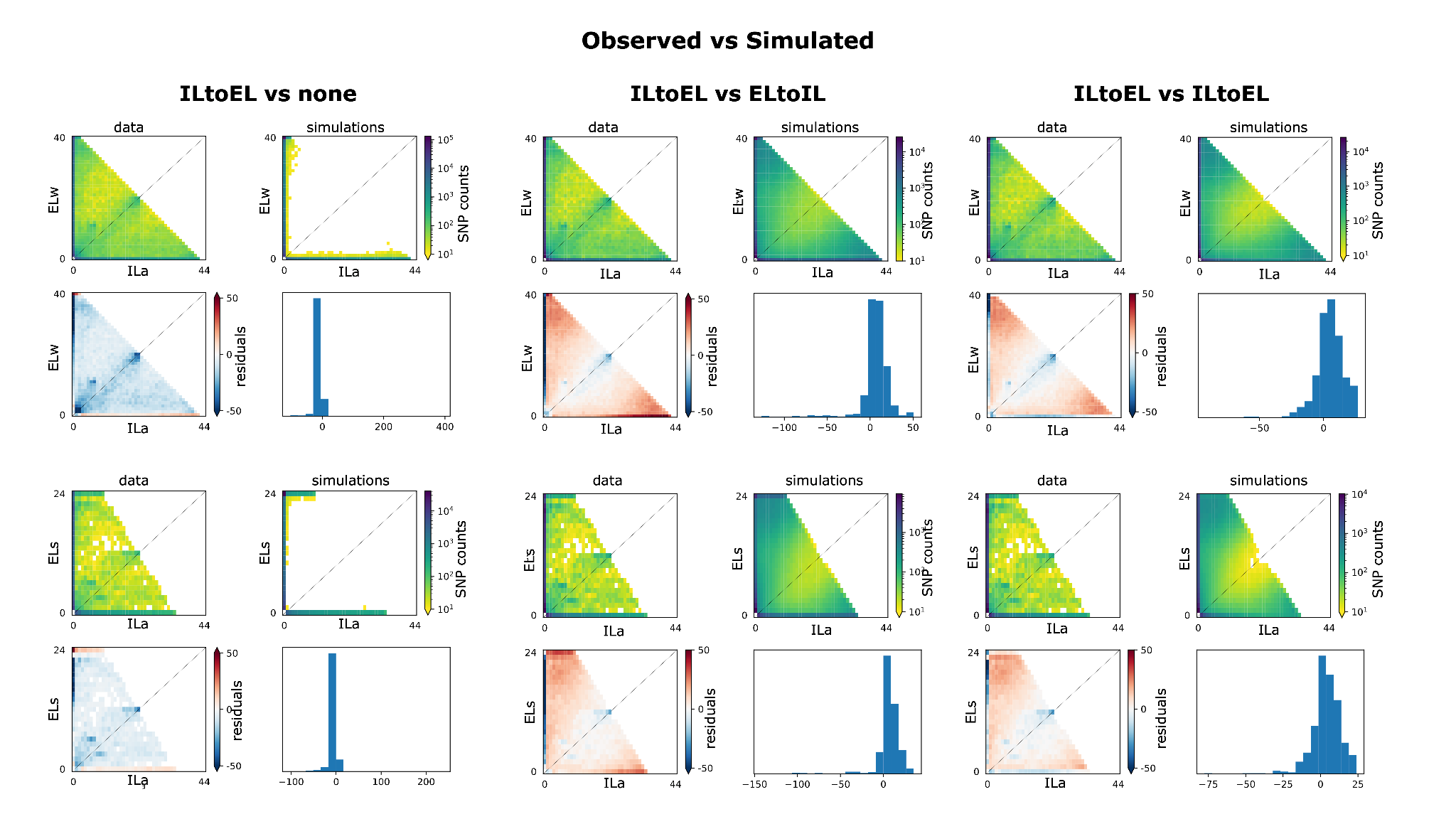
**

**Figure S11.** Two dimensional SFS comparisons between observed data with introgression into the Eurasian lynx population and training simulations run without pulse migrations (left), with ELtoIL introgression (center), and with ILtoEL introgression (right), in ILa-ELw (top) and ILa-ELw (bottom) models. Within each population pair’s panel, overall SNP counts are displayed above and Anscombe residuals from SFS cell comparisons together with overall residual distributions are displayed below. Red values indicate higher SNP counts in simulations, whereas blue values indicate higher SNP counts in real data.


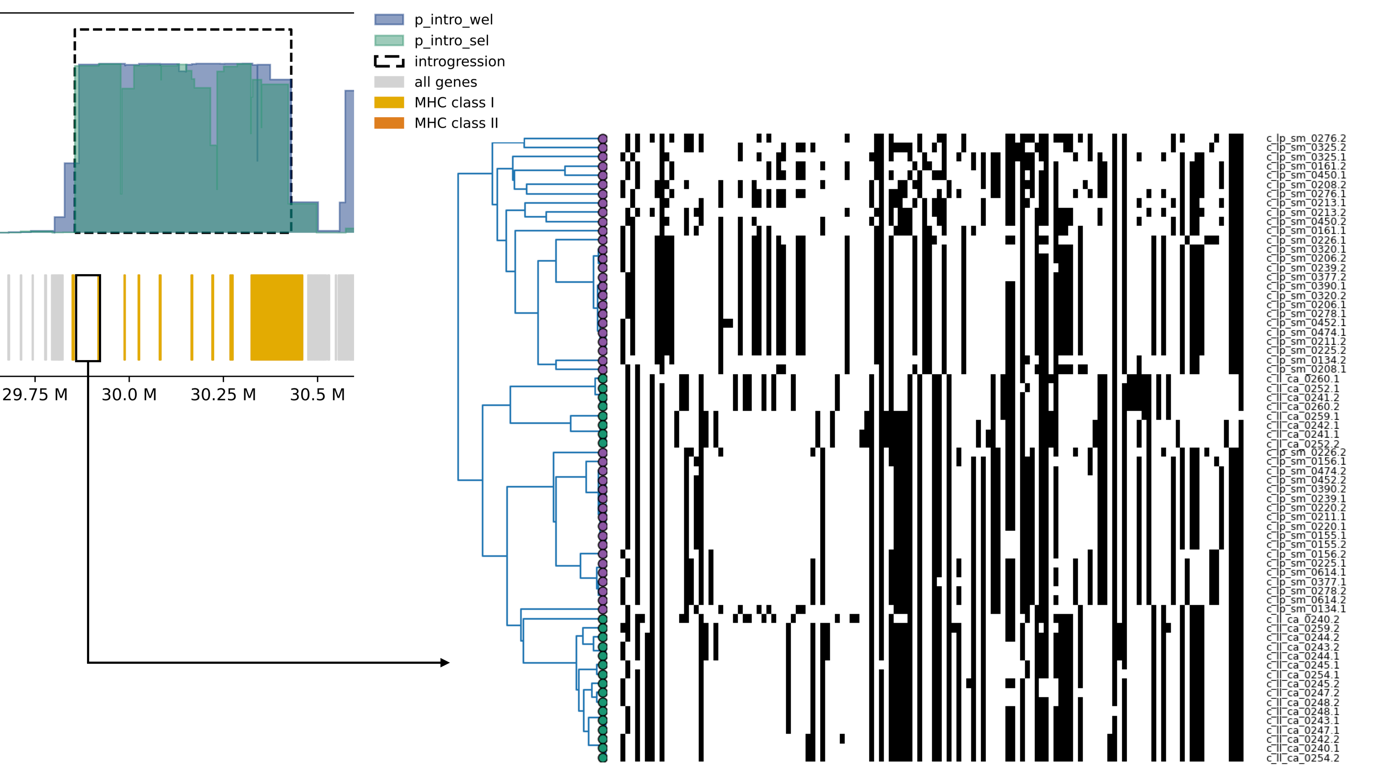


**Figure S12.** Example two population alignment matrix between ELs and ILa individuals (rows) with SNPs from the MHC class I region (columns). Cell values represent either reference (white) or alternative (black) allele. The dendrogram on the left illustrates the relationships among samples, with individual haplotypes clustered using average linkage based on a Hamming distance matrix calculated from the genotype matrix. Circles on the leaf of the dendrogram are color coded by population (ILa: violet, ELs: green). Sample names are displayed on the right.


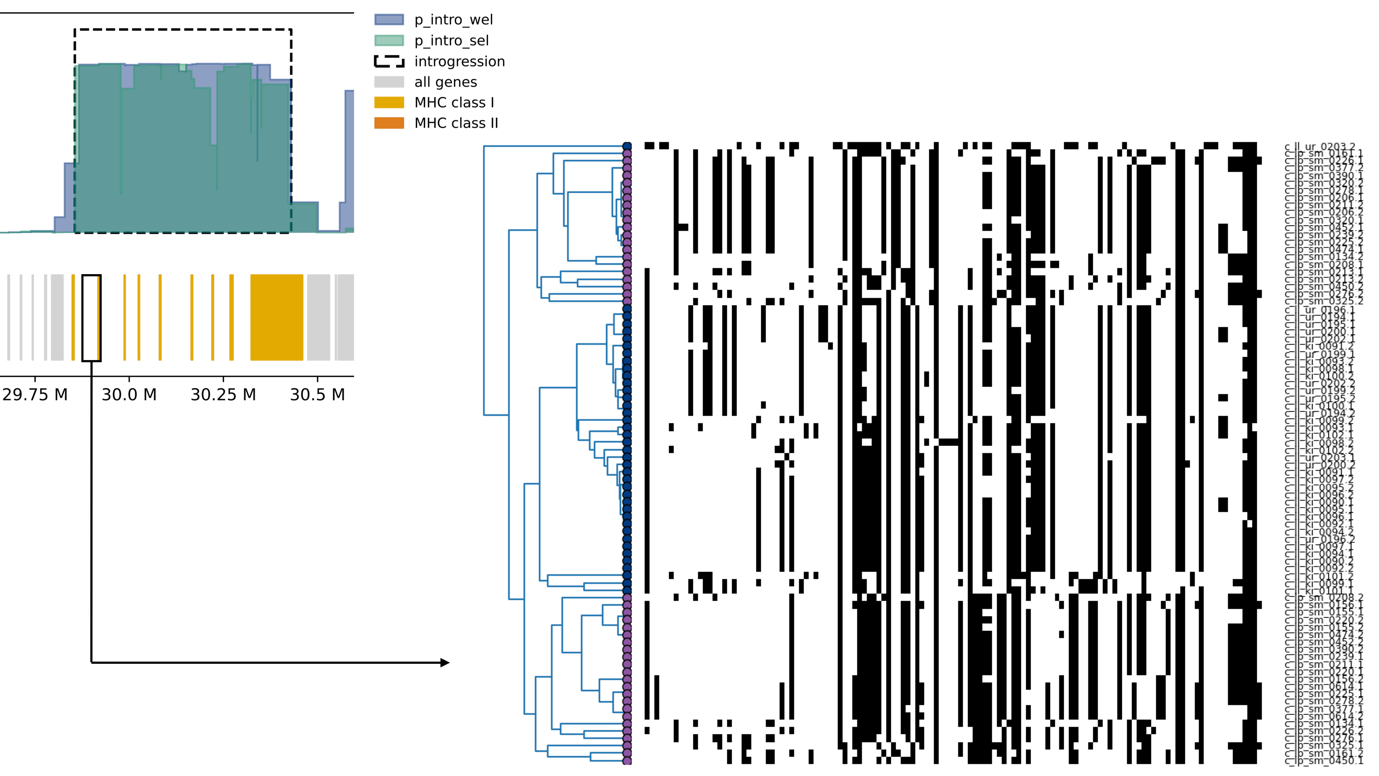


**Figure S13.** Example two population alignment matrix between ELw and ILa individuals (rows) with SNPs from the MHC class I region (columns). Cell values represent either reference (white) or alternative (black) allele. The dendrogram on the left illustrates the relationships among samples, with individual haplotypes clustered using average linkage based on a Hamming distance matrix calculated from the genotype matrix. Circles on the leaf of the dendrogram are color coded by population (ILa: violet, ELw: blue). Sample names are displayed on the right.


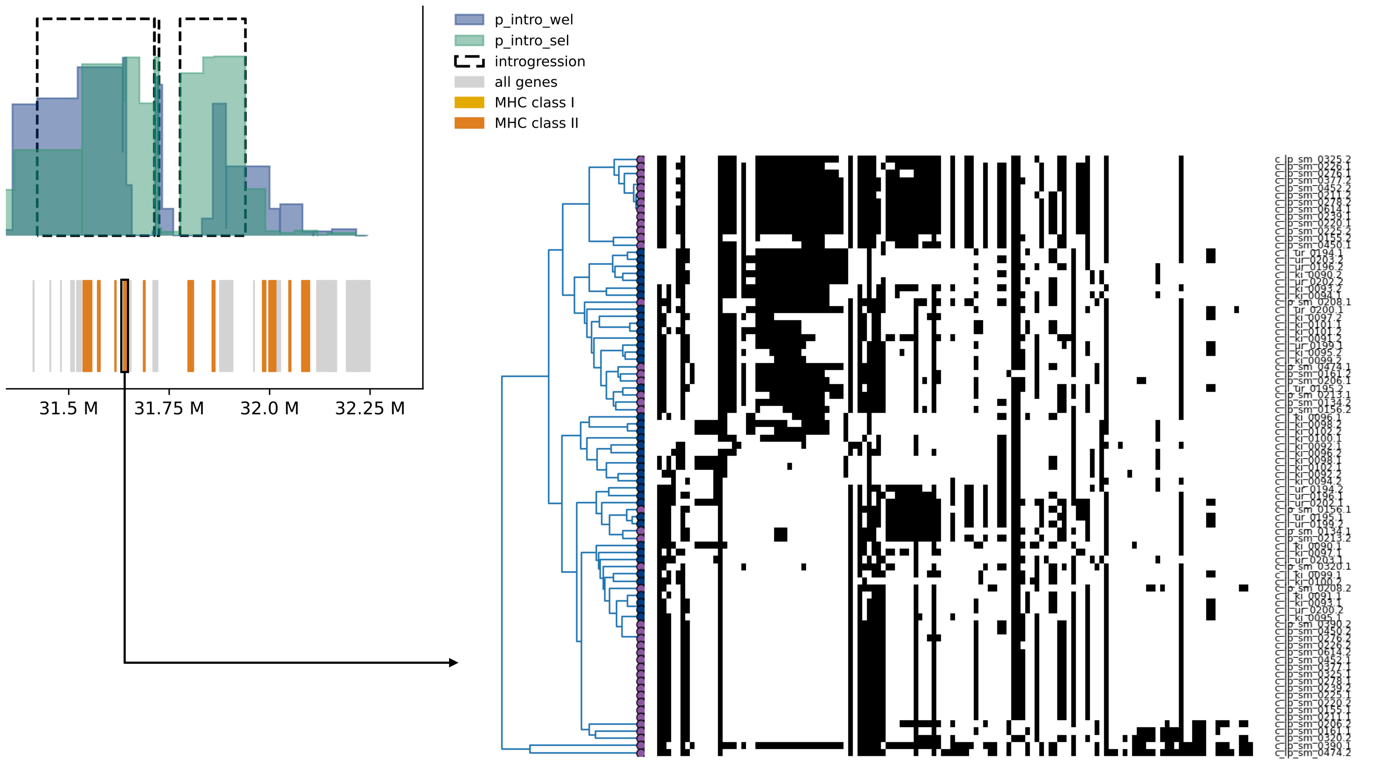


**Figure S14.** Example two population alignment matrix between ELw and ILa individuals (rows) with SNPs from the MHC class II region (columns). Cell values represent either reference (white) or alternative (black) allele. The dendrogram on the left illustrates the relationships among samples, with individual haplotypes clustered using average linkage based on a Hamming distance matrix calculated from the genotype matrix. Circles on the leaf of the dendrogram are color coded by population (ILa: violet, ELw: blue). Sample names are displayed on the right.


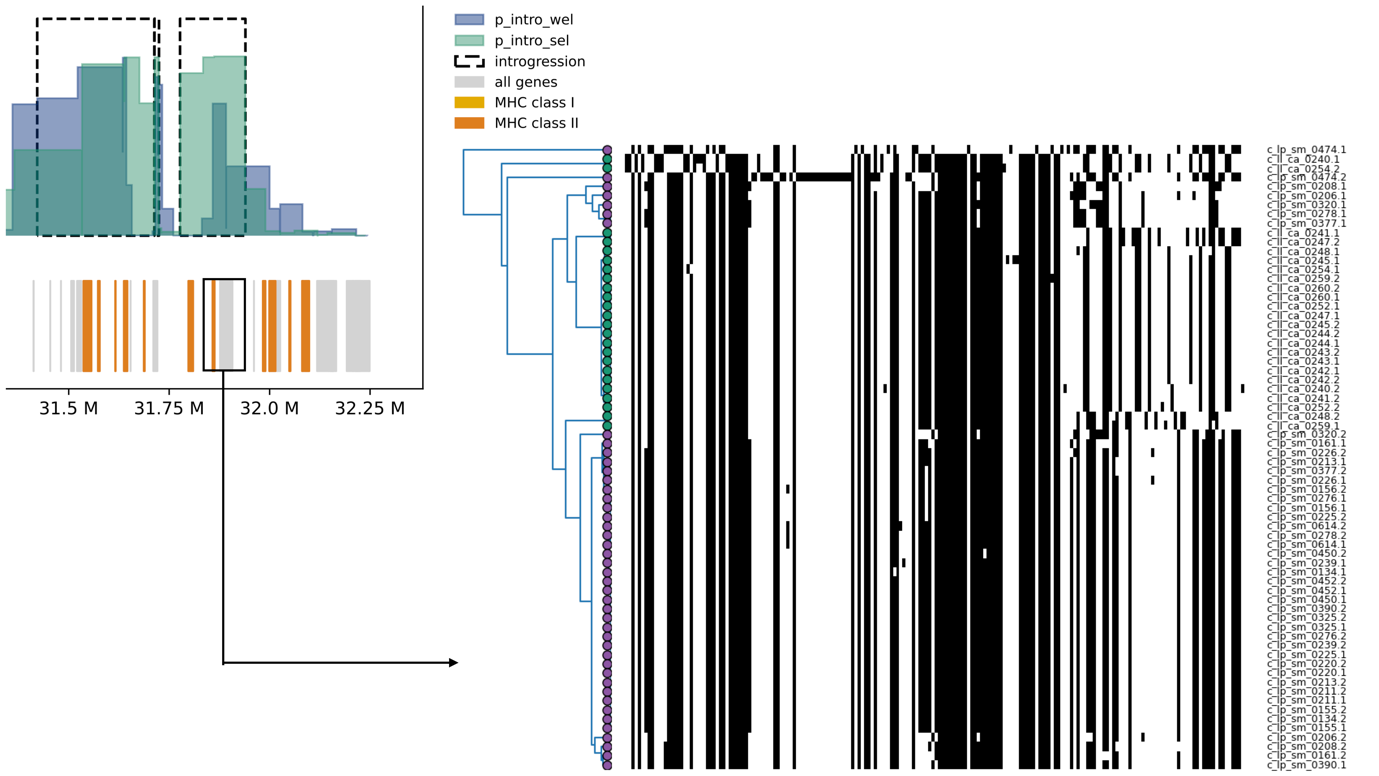


**Figure S15.** Example two population alignment matrix between ELs and ILa individuals (rows) with SNPs from the MHC class II region (columns). Cell values represent either reference (white) or alternative (black) allele. The dendrogram on the left illustrates the relationships among samples, with individual haplotypes clustered using average linkage based on a Hamming distance matrix calculated from the genotype matrix. Circles on the leaf of the dendrogram are color coded by population (ILa: violet, ELs: green). Sample names are displayed on the right.

**
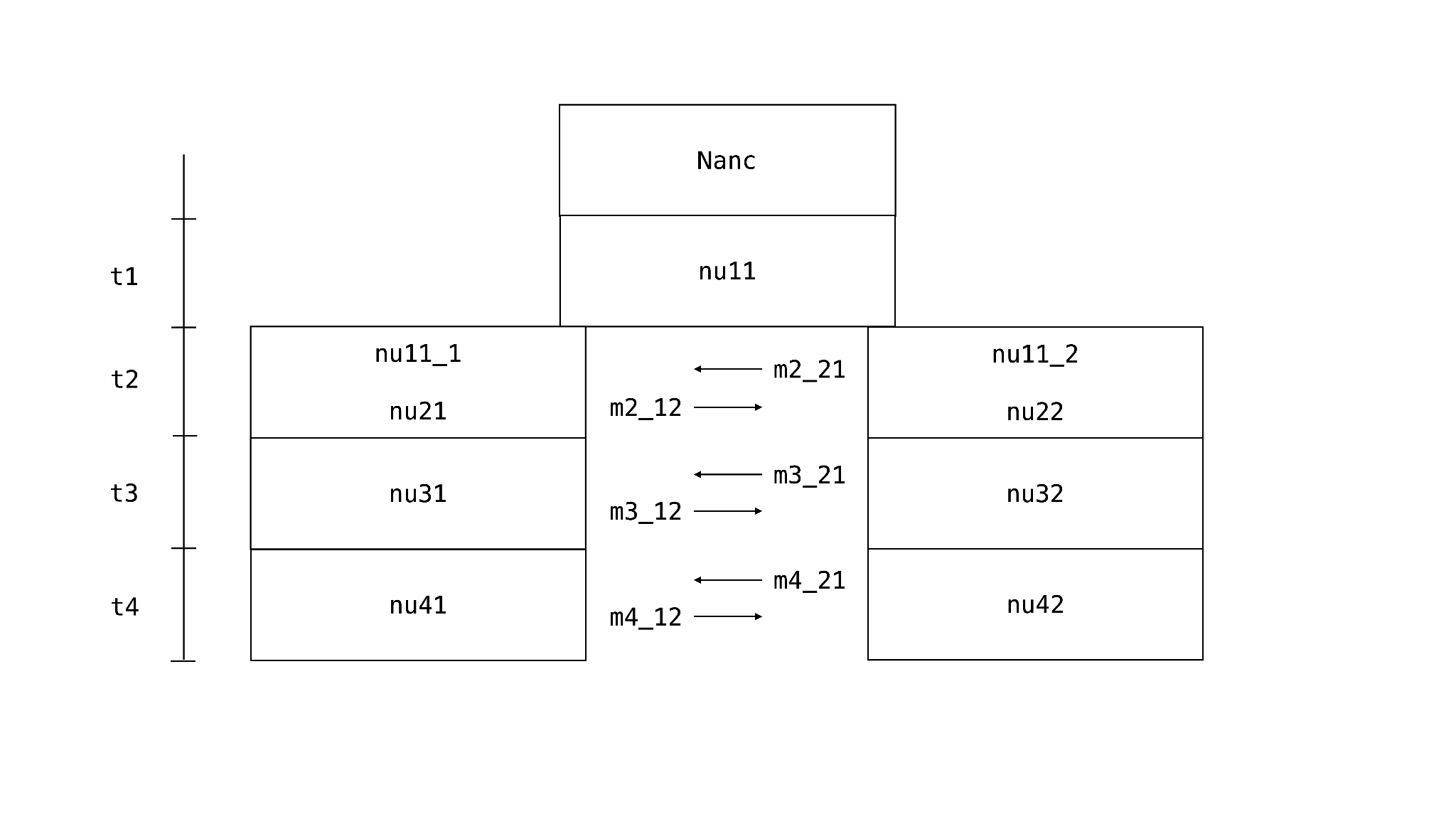
**

**Figure S16.** Basic configuration of the structured model used for demographic modeling with GADMA2. All the parameters estimated are listed, with ‘t’ parameters indicating number of generations for each epoch, ‘nu’ parameters indicating effective population size at the end of each epoch and ‘m’ parameters indicating asymmetrical migration rates in each epoch.

**Table S1.** Sampled individuals information. Sample’s name, species and population, publication in which it was first sequenced, Illumina platform used and average whole genome sequencing depth are provided.

| SAMPLE | SPECIES | POPULATION | PUBLICATION | PLATFORM | DEPTH |
| --- | --- | --- | --- | --- | --- |
| c_lp_sm_0134 | *Lynx pardinus* | Iberian lynx (LPA) | Kleinman-Ruiz et al. 2022 | HiSeq2000,v3 | 6X |
| c_lp_sm_0155 | *Lynx pardinus* | Iberian lynx (LPA) | Kleinman-Ruiz et al. 2022 | HiSeq2000,v3 | 5X |
| c_lp_sm_0156 | *Lynx pardinus* | Iberian lynx (LPA) | Kleinman-Ruiz et al. 2022 | HiSeq2000,v3 | 6X |
| c_lp_sm_0161 | *Lynx pardinus* | Iberian lynx (LPA) | Kleinman-Ruiz et al. 2022 | HiSeq2000,v3 | 5X |
| c_lp_sm_0206 | *Lynx pardinus* | Iberian lynx (LPA) | Kleinman-Ruiz et al. 2022 | HiSeq2000,v3 | 5X |
| c_lp_sm_0208 | *Lynx pardinus* | Iberian lynx (LPA) | Kleinman-Ruiz et al. 2022 | HiSeq2000,v3 | 5X |
| c_lp_sm_0213 | *Lynx pardinus* | Iberian lynx (LPA) | Kleinman-Ruiz et al. 2022 | HiSeq2000,v3 | 5X |
| c_lp_sm_0226 | *Lynx pardinus* | Iberian lynx (LPA) | Kleinman-Ruiz et al. 2022 | HiSeq2000,v3 | 5X |
| c_lp_sm_0276 | *Lynx pardinus* | Iberian lynx (LPA) | Kleinman-Ruiz et al. 2022 | HiSeq2000,v3 | 5X |
| c_lp_sm_0320 | *Lynx pardinus* | Iberian lynx (LPA) | Kleinman-Ruiz et al. 2022 | HiSeq2000,v3 | 5X |
| c_lp_sm_0325 | *Lynx pardinus* | Iberian lynx (LPA) | Kleinman-Ruiz et al. 2022 | HiSeq2000,v3 | 5X |
| c_lp_sm_0450 | *Lynx pardinus* | Iberian lynx (LPA) | Kleinman-Ruiz et al. 2022 | HiSeq2000,v3 | 5X |
| c_lp_sm_0211 | *Lynx pardinus* | Iberian lynx (LPA) | Present study | NovaSeqXPlus | 33X |
| c_lp_sm_0220 | *Lynx pardinus* | Iberian lynx (LPA) | Present study | NovaSeqXPlus | 43X |
| c_lp_sm_0225 | *Lynx pardinus* | Iberian lynx (LPA) | Present study | NovaSeqXPlus | 33X |
| c_lp_sm_0239 | *Lynx pardinus* | Iberian lynx (LPA) | Present study | NovaSeqXPlus | 40X |
| c_lp_sm_0278 | *Lynx pardinus* | Iberian lynx (LPA) | Present study | NovaSeqXPlus | 38X |
| c_lp_sm_0377 | *Lynx pardinus* | Iberian lynx (LPA) | Present study | NovaSeqXPlus | 33X |
| c_lp_sm_0390 | *Lynx pardinus* | Iberian lynx (LPA) | Present study | NovaSeqXPlus | 32X |
| c_lp_sm_0452 | *Lynx pardinus* | Iberian lynx (LPA) | Present study | NovaSeqXPlus | 34X |
| c_lp_sm_0474 | *Lynx pardinus* | Iberian lynx (LPA) | Present study | NovaSeqXPlus | 40X |
| c_lp_sm_0614 | *Lynx pardinus* | Iberian lynx (LPA) | Present study | NovaSeqXPlus | 53X |
| c_ll_ca_0240 | *Lynx lynx* | Southern Eurasian lynx (SEL) | Bazzicalupo et al. 2022 | HiSeq2000,v4 | 39X |
| c_ll_ca_0241 | *Lynx lynx* | Southern Eurasian lynx (SEL) | Bazzicalupo et al. 2022 | HiSeq2000,v4 | 24X |
| c_ll_ca_0242 | *Lynx lynx* | Southern Eurasian lynx (SEL) | Bazzicalupo et al. 2022 | HiSeq2000,v4 | 24X |
| c_ll_ca_0243 | *Lynx lynx* | Southern Eurasian lynx (SEL) | Bazzicalupo et al. 2022 | HiSeq2000,v4 | 29X |
| c_ll_ca_0244 | *Lynx lynx* | Southern Eurasian lynx (SEL) | Bazzicalupo et al. 2022 | HiSeq2000,v4 | 20X |
| c_ll_ca_0245 | *Lynx lynx* | Southern Eurasian lynx (SEL) | Bazzicalupo et al. 2022 | HiSeq2000,v4 | 10X |
| c_ll_ca_0247 | *Lynx lynx* | Southern Eurasian lynx (SEL) | Bazzicalupo et al. 2022 | HiSeq2000,v4 | 14X |
| c_ll_ca_0248 | *Lynx lynx* | Southern Eurasian lynx (SEL) | Bazzicalupo et al. 2022 | HiSeq2000,v4 | 12X |
| c_ll_ca_0252 | *Lynx lynx* | Southern Eurasian lynx (SEL) | Bazzicalupo et al. 2022 | HiSeq2000,v4 | 18X |
| c_ll_ca_0254 | *Lynx lynx* | Southern Eurasian lynx (SEL) | Bazzicalupo et al. 2022 | HiSeq2000,v4 | 15X |
| c_ll_ca_0259 | *Lynx lynx* | Southern Eurasian lynx (SEL) | Bazzicalupo et al. 2022 | HiSeq2000,v4 | 13X |
| c_ll_ca_0260 | *Lynx lynx* | Southern Eurasian lynx (SEL) | Bazzicalupo et al. 2022 | HiSeq2000,v4 | 17X |
| c_ll_ki_0090 | *Lynx lynx* | Western Eurasian lynx (SEL) | Lucena-Perez et al. 2020 | HiSeq X-10 | 23X |
| c_ll_ki_0091 | *Lynx lynx* | Western Eurasian lynx (SEL) | Lucena-Perez et al. 2020 | HiSeq2000,v3 | 6X |
| c_ll_ki_0092 | *Lynx lynx* | Western Eurasian lynx (SEL) | Lucena-Perez et al. 2020 | HiSeq2000,v3 | 5X |
| c_ll_ki_0093 | *Lynx lynx* | Western Eurasian lynx (SEL) | Lucena-Perez et al. 2020 | HiSeq2000,v3 | 5X |
| c_ll_ki_0094 | *Lynx lynx* | Western Eurasian lynx (SEL) | Lucena-Perez et al. 2020 | HiSeq2000,v3 | 6X |
| c_ll_ki_0095 | *Lynx lynx* | Western Eurasian lynx (SEL) | Lucena-Perez et al. 2020 | HiSeq2000,v3 | 5X |
| c_ll_ki_0096 | *Lynx lynx* | Western Eurasian lynx (SEL) | Lucena-Perez et al. 2020 | HiSeq2000,v3 | 6X |
| c_ll_ki_0097 | *Lynx lynx* | Western Eurasian lynx (SEL) | Lucena-Perez et al. 2020 | HiSeq2000,v3 | 6X |
| c_ll_ki_0098 | *Lynx lynx* | Western Eurasian lynx (SEL) | Lucena-Perez et al. 2020 | HiSeq2000,v3 | 6X |
| c_ll_ki_0099 | *Lynx lynx* | Western Eurasian lynx (SEL) | Lucena-Perez et al. 2020 | HiSeq2000,v3 | 6X |
| c_ll_ki_0100 | *Lynx lynx* | Western Eurasian lynx (SEL) | Lucena-Perez et al. 2020 | HiSeq2000,v3 | 6X |
| c_ll_ki_0101 | *Lynx lynx* | Western Eurasian lynx (SEL) | Lucena-Perez et al. 2020 | HiSeq2000,v3 | 6X |
| c_ll_ki_0102 | *Lynx lynx* | Western Eurasian lynx (SEL) | Lucena-Perez et al. 2020 | HiSeq2000,v3 | 6X |
| c_ll_ur_0194 | *Lynx lynx* | Western Eurasian lynx (SEL) | Lucena-Perez et al. 2020 | HiSeq2000,v4 | 11X |
| c_ll_ur_0195 | *Lynx lynx* | Western Eurasian lynx (SEL) | Lucena-Perez et al. 2020 | HiSeq2000,v4 | 12X |
| c_ll_ur_0196 | *Lynx lynx* | Western Eurasian lynx (SEL) | Lucena-Perez et al. 2020 | HiSeq2000,v4 | 12X |
| c_ll_ur_0199 | *Lynx lynx* | Western Eurasian lynx (SEL) | Lucena-Perez et al. 2020 | HiSeq2000,v4 | 13X |
| c_ll_ur_0200 | *Lynx lynx* | Western Eurasian lynx (SEL) | Lucena-Perez et al. 2020 | HiSeq2000,v4 | 12X |
| c_ll_ur_0202 | *Lynx lynx* | Western Eurasian lynx (SEL) | Bazzicalupo et al. 2023 | HiSeq2000,v4 | 25X |
| c_ll_ur_0203 | *Lynx lynx* | Western Eurasian lynx (SEL) | Lucena-Perez et al. 2020 | HiSeq2000,v4 | 13X |

**Table S2.** Enrichment results for Biological Process GO-terms of genes found in introgressed regions of Iberian lynx (ILa). GO-terms with a Fisher exact test p-values < 0.01 are reported.

| **GO.ID** | **Term** | **Annotated** | **Significant** | **Expected** | **Fisher** |
| --- | --- | --- | --- | --- | --- |
| **GO:0019882** | antigen processing and presentation | 60 | 19 | 5.47 | 0.00010 |
| **GO:0019731** | antibacterial humoral response | 24 | 9 | 2.19 | 0.00016 |
| **GO:0006334** | nucleosome assembly | 54 | 14 | 4.92 | 0.00025 |
| **GO:0070588** | calcium ion transmembrane transport | 194 | 22 | 17.68 | 0.00121 |
| **GO:0007606** | sensory perception of chemical stimulus | 862 | 147 | 78.56 | 0.00303 |
| **GO:0097350** | neutrophil clearance | 8 | 4 | 0.73 | 0.00356 |
| **GO:0002503** | peptide antigen assembly with MHC class II protein complex | 8 | 4 | 0.73 | 0.00356 |
| **GO:0006935** | chemotaxis | 342 | 30 | 31.17 | 0.00555 |
| **GO:0019886** | antigen processing and presentation of exogenous peptide antigen via MHC class II | 9 | 4 | 0.82 | 0.00595 |
| **GO:0002227** | innate immune response in mucosa | 9 | 4 | 0.82 | 0.00595 |
| **GO:0019732** | antifungal humoral response | 5 | 3 | 0.46 | 0.00656 |
| **GO:0061049** | cell growth involved in cardiac muscle cell development | 6 | 4 | 0.55 | 0.00828 |
| **GO:0036114** | medium-chain fatty-acyl-CoA catabolic process | 2 | 2 | 0.18 | 0.00830 |
| **GO:0005986** | sucrose biosynthetic process | 2 | 2 | 0.18 | 0.00830 |
| **GO:2001294** | malonyl-CoA catabolic process | 2 | 2 | 0.18 | 0.00830 |
| **GO:0044580** | butyryl-CoA catabolic process | 2 | 2 | 0.18 | 0.00830 |
| **GO:0015835** | peptidoglycan transport | 2 | 2 | 0.18 | 0.00830 |
| **GO:0071866** | negative regulation of apoptotic process in bone marrow cell | 2 | 2 | 0.18 | 0.00830 |
| **GO:0002491** | antigen processing and presentation of endogenous peptide antigen via MHC class II | 2 | 2 | 0.18 | 0.00830 |
| **GO:0002469** | myeloid dendritic cell antigen processing and presentation | 2 | 2 | 0.18 | 0.00830 |
| **GO:0015938** | coenzyme A catabolic process | 2 | 2 | 0.18 | 0.00830 |
| **GO:1901289** | succinyl-CoA catabolic process | 2 | 2 | 0.18 | 0.00830 |
| **GO:1901248** | positive regulation of lung ciliated cell differentiation | 2 | 2 | 0.18 | 0.00830 |
| **GO:0045870** | positive regulation of single stranded viral RNA replication via double stranded DNA intermediate | 2 | 2 | 0.18 | 0.00830 |
| **GO:1903861** | positive regulation of dendrite extension | 10 | 4 | 0.91 | 0.00921 |

**Table S3.** Enrichment results for Biological Process GO-terms of genes found in introgressed regions of Western Eurasian lynx (ELw). GO-terms with a Fisher exact test p-values < 0.01 are reported.

| **GO.ID** | **Term** | **Annotated** | **Significant** | **Expected** | **Fisher** |
| --- | --- | --- | --- | --- | --- |
| **GO:0051923** | sulfation | 15 | 6 | 1.26 | 0.00073 |
| **GO:0007606** | sensory perception of chemical stimulus | 862 | 82 | 72.47 | 0.00261 |
| **GO:0010954** | positive regulation of protein processing | 13 | 5 | 1.09 | 0.00263 |
| **GO:0016339** | calcium-dependent cell-cell adhesion via plasma membrane cell adhesion molecules | 13 | 5 | 1.09 | 0.00302 |
| **GO:1900748** | positive regulation of vascular endothelial growth factor signaling pathway | 5 | 3 | 0.42 | 0.00521 |
| **GO:0044346** | fibroblast apoptotic process | 19 | 4 | 1.6 | 0.00521 |
| **GO:0150003** | regulation of spontaneous synaptic transmission | 4 | 3 | 0.34 | 0.00705 |
| **GO:0016199** | axon midline choice point recognition | 2 | 2 | 0.17 | 0.00706 |
| **GO:0055111** | ingression involved in gastrulation with mouth forming second | 2 | 2 | 0.17 | 0.00706 |
| **GO:1903660** | negative regulation of complement-dependent cytotoxicity | 2 | 2 | 0.17 | 0.00706 |
| **GO:0046544** | development of secondary male sexual characteristics | 2 | 2 | 0.17 | 0.00706 |
| **GO:1900272** | negative regulation of long-term synaptic potentiation | 2 | 2 | 0.17 | 0.00706 |
| **GO:1905931** | negative regulation of vascular associated smooth muscle cell differentiation involved in phenotypic switching | 2 | 2 | 0.17 | 0.00706 |
| **GO:0010750** | positive regulation of nitric oxide mediated signal transduction | 2 | 2 | 0.17 | 0.00706 |
| **GO:0034463** | 90S preribosome assembly | 2 | 2 | 0.17 | 0.00706 |
| **GO:0071403** | cellular response to high density lipoprotein particle stimulus | 2 | 2 | 0.17 | 0.00706 |
| **GO:1901485** | positive regulation of transcription factor catabolic process | 2 | 2 | 0.17 | 0.00706 |
| **GO:0009590** | detection of gravity | 2 | 2 | 0.17 | 0.00706 |
| **GO:0030178** | negative regulation of Wnt signaling pathway | 109 | 15 | 9.16 | 0.00798 |
| **GO:0010803** | regulation of tumor necrosis factor-mediated signaling pathway | 27 | 7 | 2.27 | 0.00971 |
| **GO:0002504** | antigen processing and presentation of peptide or polysaccharide antigen via MHC class II | 19 | 6 | 1.6 | 0.00972 |
| **GO:1901526** | positive regulation of mitophagy | 6 | 3 | 0.5 | 0.00976 |
| **GO:0034351** | negative regulation of glial cell apoptotic process | 6 | 3 | 0.5 | 0.00976 |

**Table S4.** Enrichment results for Biological Process GO-terms of genes found in introgressed regions of Southern Eurasian lynx (ELs). GO-terms with a Fisher exact test p-values < 0.01 are reported.

| **GO.ID** | **Term** | **Annotated** | **Significant** | **Expected** | **Fisher** |
| --- | --- | --- | --- | --- | --- |
| **GO:0150003** | regulation of spontaneous synaptic transmission | 4 | 3 | 0.12 | 0.00097 |
| **GO:0016255** | attachment of GPI anchor to protein | 2 | 2 | 0.06 | 0.00097 |
| **GO:1900272** | negative regulation of long-term synaptic potentiation | 2 | 2 | 0.06 | 0.00097 |
| **GO:0010750** | positive regulation of nitric oxide mediated signal transduction | 2 | 2 | 0.06 | 0.00097 |
| **GO:0097009** | energy homeostasis | 26 | 5 | 0.81 | 0.00111 |
| **GO:0043046** | DNA methylation involved in gamete generation | 8 | 3 | 0.25 | 0.00150 |
| **GO:0034587** | piRNA metabolic process | 9 | 3 | 0.28 | 0.00220 |
| **GO:0010636** | positive regulation of mitochondrial fusion | 3 | 2 | 0.09 | 0.00285 |
| **GO:0038060** | nitric oxide-cGMP-mediated signaling pathway | 3 | 2 | 0.09 | 0.00285 |
| **GO:0034727** | piecemeal microautophagy of the nucleus | 3 | 2 | 0.09 | 0.00285 |
| **GO:1990504** | dense core granule exocytosis | 3 | 2 | 0.09 | 0.00285 |
| **GO:0016322** | neuron remodeling | 10 | 3 | 0.31 | 0.00307 |
| **GO:0021540** | corpus callosum morphogenesis | 4 | 2 | 0.12 | 0.00559 |
| **GO:0036150** | phosphatidylserine acyl-chain remodeling | 4 | 2 | 0.12 | 0.00559 |
| **GO:0036152** | phosphatidylethanolamine acyl-chain remodeling | 4 | 2 | 0.12 | 0.00559 |
| **GO:0019441** | tryptophan catabolic process to kynurenine | 4 | 2 | 0.12 | 0.00559 |
| **GO:0006182** | cGMP biosynthetic process | 14 | 3 | 0.44 | 0.00850 |
| **GO:0060087** | relaxation of vascular associated smooth muscle | 5 | 2 | 0.16 | 0.00913 |
| **GO:0021960** | anterior commissure morphogenesis | 5 | 2 | 0.16 | 0.00913 |
| **GO:1900122** | positive regulation of receptor binding | 5 | 2 | 0.16 | 0.00913 |
